# Lithium–Sulfur Batteries Meet Electrospinning: Recent Advances and the Key Parameters for High Gravimetric and Volume Energy Density

**DOI:** 10.1002/advs.202103879

**Published:** 2021-11-18

**Authors:** Yongshang Zhang, Xilai Zhang, S. Ravi P. Silva, Bin Ding, Peng Zhang, Guosheng Shao

**Affiliations:** ^1^ State Center for International Cooperation on Designer Low‐Carbon & Environmental Materials (CDLCEM) School of Materials Science and Engineering 100 Kexue Avenue Zhengzhou University Zhengzhou 450001 China; ^2^ Zhengzhou Materials Genome Institute (ZMGI) Xingyang Zhengzhou 450100 China; ^3^ Nanoelectronics Center Advanced Technology Institute University of Surrey Guildford GU2 7XH UK; ^4^ State Key Laboratory for Modification of Chemical Fibers and Polymer Materials College of Textile Donghua University Shanghai 201620 China

**Keywords:** electrospinning, energy density, key parameters, Li–S batteries, mathematic model

## Abstract

Lithium–sulfur (Li–S) batteries have been regarded as a promising next‐generation energy storage technology for their ultrahigh theoretical energy density compared with those of the traditional lithium‐ion batteries. However, the practical applications of Li–S batteries are still blocked by notorious problems such as the shuttle effect and the uncontrollable growth of lithium dendrites. Recently, the rapid development of electrospinning technology provides reliable methods in preparing flexible nanofibers materials and is widely applied to Li–S batteries serving as hosts, interlayers, and separators, which are considered as a promising strategy to achieve high energy density flexible Li–S batteries. In this review, a fundamental introduction of electrospinning technology and multifarious electrospinning‐based nanofibers used in flexible Li–S batteries are presented. More importantly, crucial parameters of specific capacity, electrolyte/sulfur (E/S) ratio, sulfur loading, and cathode tap density are emphasized based on the proposed mathematic model, in which the electrospinning‐based nanofibers are used as important components in Li–S batteries to achieve high gravimetric (*W*_G_) and volume (*W*_V_) energy density of 500 Wh kg^−1^ and 700 Wh L^−1^, respectively. These systematic summaries not only provide the principles in nanofiber‐based electrode design but also propose enlightening directions for the commercialized Li–S batteries with high *W*_G_ and *W*_V_.

## Graphical abstract

A mathematic model is proposed using the electrospinning‐based nanofibers serve as a cathode and anode host, and separator to achieve high gravimetric (*W*_G_) and volume (*W*_V_) energy density of 500 Wh kg^−1^ and 700 Wh L^−1^ in Li–S batteries, respectively, by emphasizing the crucial parameters of specific capacity, electrolyte/sulfur (E/S) ratio, sulfur loading, and cathode tap density in the cell.

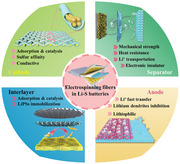

## 1 Introduction

With the rapid depletion of fossil resources and increasing environmental pollution, environment‐friendly renewable energy sources are urgently needed. Since the exploitation of common renewable energy sources (wind, geothermal, and solar) are limited by their intermittent nature, the development of highly efficient energy storage systems with high gravimetric (*W*_G_) and volume (*W*_V_) energy density have been under intense investigation in recent years.^[^ ^1^ ^]^

As one of the most prospective candidates, lithium–sulfur (Li–S) batteries have been intensively studied, owing to their low cost, high theoretical capacity (1675 mAh g^−1^) and energy density (2600 Wh kg^−1^), and as well as environmental friendliness.^[^ ^2^ ^]^ However, the complex multi‐step electrochemical reaction process and solid‐liquid‐solid phase conversion between S and Li_2_S lead to severely inherent limits for the practical applications of Li–S batteries.^[^ ^3^ ^]^ What is more, the notorious issues including poor conductivity of S and Li_2_S,^[^ ^4^ ^]^ shuttle effect of polysulfides (LiPS)^[^ ^5^ ^]^ and sluggish conversion kinetics of sulfur species in the cathode,^[^ ^6^ ^]^ the overgrowth of lithium dendrites,^[^ ^7^ ^]^ and unstable solid electrolyte interface (SEI) in the anode,^[^ ^8^ ^]^ and high flammability of the separator^[^ ^9^ ^]^ could also cause sluggish reaction kinetics, insufficient sulfur utilization, poor cycle stability, and safety hazards in the Li–S batteries. It is of great significance and challenge to solve the above problems as a whole.^[^ ^10^ ^]^

In the last decades, many researchers have dedicated themselves to developing novel technologies and materials to solve the problems in Li–S batteries.^[^ ^11^ ^]^ Benefiting from this, the electrochemical performance of Li–S batteries has been enhanced to a better level and the involved reaction mechanism is becoming clear.^[^ ^12^ ^]^ Based on the problematic issues in the cathode, anode, and separator of Li–S batteries, many types of functional materials and structures have been constructed and modified.^[^ ^13^ ^]^ Among them, the adoption of various nanofiber structural materials in the Li–S batteries has been considered as a promising strategy, due to the unique structure and compositional diversity. As a common method in preparing nanofibers, the electrospinning technology shows many merits such as simple, versatile, and low cost. And, the electrospun nanofibers could be designed by adjusting the components of precursor or subsequent procedure to obtain unique structures (nanowires, hollow, yolk–shell, multi‐yolk–shell, and composite structure) and multiple functional groups (self‐standing, flexible, high mechanical strength, and large surface area), which make them suitable applied to Li–S batteries serving as hosts, interlayers, and separators.

As the most important component of the Li–S battery system, the cathode, has suffered a lot from the poor conductivity, shuttle effect,^[^ ^14^ ^]^ sluggish reaction kinetics, and volume fluctuation between S and Li_2_S,^[^ ^15^ ^]^ which could be resolved by using electrospinning derived carbon nanofibers coupling with electrocatalysts serve as sulfur host.^[^ ^16^ ^]^ On the other hand, the separator is also a fundamental and critical part of the Li–S battery system, which generally is a porous polymer membrane (such as polyethylene (PE) and polypropylene (PP)) and easy to transport lithium ions but electrical insulation.^[^ ^17^ ^]^ Since the current PP/PE separators have good applicability and stability in the Li–S battery, they also have some serious shortcomings: 1) inferior thermal stability; 2) poor electrolyte wettability; 3) poor polysulfides barrier ability.^[^ ^18^ ^]^ Electrospinning‐based nanofibers could also deal with the obstacles the PP/PE separators meet in the Li–S batteries. For example, Mai's group reported a modified electrospinning separator, which possessed the merits of good mechanical property, thermal stability, and restrained LiPS shuttle, enabling the outstanding electrochemical performance of Li–S batteries.^[^ ^19^ ^]^ As for the lithium anode, the overgrowth of Li dendrites could be confined by using electrospinning derived 3 D frameworks to serve as lithiophilic hosts, which have been proved in many published works.^[^ ^20^ ^]^ In short, a tremendous development can be created when Li–S batteries meet electrospinning.^[^ ^21^ ^]^

**Figure 1:**
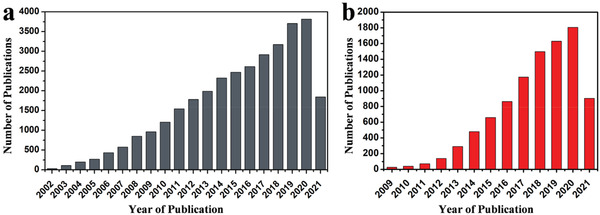
a) Statistics of publications based on electrospinning technique from 1 January 2002 to 1 July 2021 by searching “electrospinning” as “topic” in the website of Web of Science. b) Statistics of publications of Li–S batteries from 1 January 2009 to 1 July 2021 by searching “Li–S batteries” as “topic” in the website of Web of Science.

**Figure 2:**
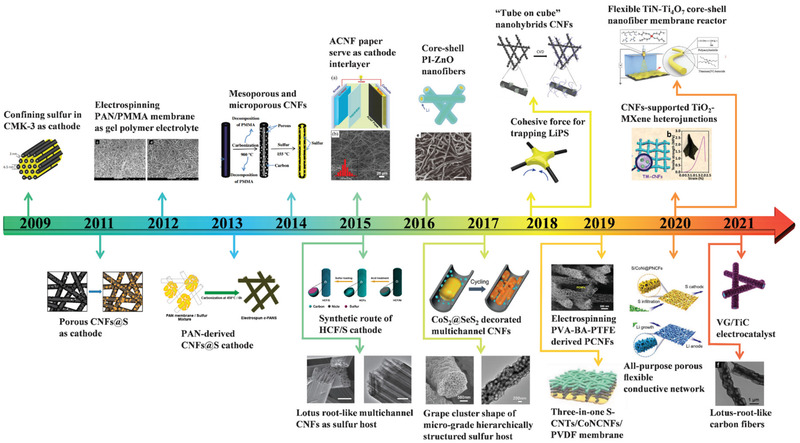
A brief timeline and representative structure based on electrospinning technique for the improvement of electrochemical performance of Li–S batteries, containing sulfur cathode host, interlayer, separator, and lithium anode host. Inserted represented works containing: The revival of Li–S batteries starts from Nazar group's work of confining sulfur in CMK‐3 as a cathode. Reproduced with permission.^[^ ^2a^ ^]^ Copyright 2009, Springer Nature. Carbonizing electrospinning PAN/PMMA as sulfur host. Reproduced with permission.^[^ ^23^ ^]^ Copyright 2011, Royal Society of Chemistry. Electrospinning PAN/PMMA membrane as gel polymer electrolyte. Reproduced with permission.^[^ ^24^ ^]^ Copyright 2012, Elsevier. Carbonization PAN nanofibers serve as sulfur cathode hosts. Reproduced with permission.^[^ ^25^ ^]^ Copyright 2013, American Chemical Society. Mesoporous and microporous CNFs derived from carbonization PAN/PMMA nanofibers. Reproduced with permission.^[^ ^26^ ^]^ Copyright 2014, Elsevier. ACNF paper serves as a cathode interlayer. Reproduced with permission.^[^ ^27^ ^]^ Copyright 2015, Elsevier. Ni nanoparticles serve as hard templates for a hierarchical structure carbon/sulfur cathode. Reproduced with permission.^[^ ^28^ ^]^ Copyright 2015, Elsevier. Lotus root‐like multichannel CNFs as sulfur host. Reproduced with permission.^[^ ^29^ ^]^ Copyright 2015, Springer Nature. Core–shell PI‐ZnO nanofibers serve as lithium anode hosts. Reproduced with permission.^[^ ^30^ ^]^ Copyright 2016, Springer Nature. CoS2@SeS2 decorated multichannel CNFs serve as sulfur hosts.^[^ ^31^ ^]^ Copyright 2017, Wiley‐VCH. Grape cluster shape of the micrograde hierarchically structured sulfur host. Reproduced with permission.^[^ ^16d^ ^]^ Copyright 2017, Elsevier. The cohesive force from the CNFs for the trapping of LiPS. Reproduced with permission.^[^ ^32^ ^]^ Copyright 2018, American Chemical Society. The “tube on cube” nanohybrids CNFs for the sulfur cathode. Reproduced with permission.^[^ ^33^ ^]^ Copyright 2018, Royal Society of Chemistry. Electrospinning PVA‐BA‐PTFE derived PCNFs. Reproduced with permission.^[^ ^34^ ^]^ Copyright 2019, Springer Nature. The three‐in‐one S‐CNTs/CoNCNFs/PVDF membrane. Reproduced with permission.^[^ ^35^ ^]^ Copyright 2019, Wiley‐VCH. All‐purpose porous flexible conductive networks serve as sulfur hosts and lithium hosts. Reproduced with permission.^[^ ^36^ ^]^ Copyright 2020, Wiley‐VCH. The CNF‐supported TiO_2_–MXene heterojunctions for sulfur cathode host. Reproduced with permission.^[^ ^37^ ^]^ Copyright 2020, Royal Society of Chemistry. Flexible TiN–Ti_4_O_7_ core–shell nanofiber membrane reactor. Reproduced with permission.^[^ ^38^ ^]^ Copyright 2020, Wiley‐VCH. The VG/TiC electrocatalyst was prepared by electrospinning coupled with the CVD method. Reproduced with permission.^[^ ^39^ ^]^ Copyright 2021, Wiley‐VCH. Lotus‐root‐like structure as lithium anode. Reproduced with permission.^[^ ^40^ ^]^ Copyright 2021, Wiley‐VCH.

Benefitting from the development of electrospinning technology, plentiful novel functional nanofiber materials emerge, providing new approaches for solving the problems of lithium‐sulfur batteries. **Figure** **1**a reveals the number of publications based on electrospinning technique drastically increasing year by year, same with the Li–S batteries (Figure 1b), which confirms the vital role of the electrospinning technique in the field of scientific research and the rapid development of lithium–sulfur batteries.^[^ ^22^ ^]^ Given the great promises of the nanofibers utilizing, this review presents the recent progress of nanofibers utilizing based on electrospinning technology in Li–S batteries, mainly including cathode sulfur host, interlayer, separator, and anode host. First, **Figure** **2** summarizes a brief timeline and representative structure based on electrospinning technique for the improvement of electrochemical performance of Li–S batteries, containing sulfur cathode host, interlayer, separator, and lithium anode host. We then give an overview of the superiority of using electrospinning technique‐based nanofibers in Li–S batteries, confirming the special role electrospinning played in response to the problems of the Li–S system. After that, a summary of the recent Li–S battery development based on electrospinning‐based nanofibers is given. More importantly, we establish a mathematical model to probe the key parameters for high energy density Li–S batteries and deduct the possible parameters using the electrospinning‐based nanofibers as important components in Li–S batteries to achieve the high *W*_G_ and *W*_V_ of 500 Wh kg^−1^ and 700 Wh L^−1^, respectively. We genuinely hope this review will enlighten the researchers with interest and passion to develop high‐energy‐density Li–S batteries in the future.

## 2 The Working Mechanism and the Main Barriers of Li–S Batteries

### 2.1 Working Mechanism

**Figure 3:**
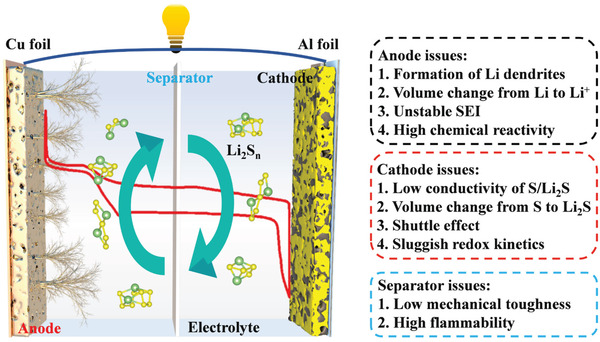
The schematic illustration of the Li–S batteries and the challenges of anode, cathode, and separator. The inside red line is the charge–discharge curve of Li–S batteries.

Li–S batteries involve multielectron reactions and multi‐phase conversion in the redox process, which makes them more complex than traditional Li‐ion batteries.^[^ ^41^ ^]^ In the past decades, many efforts have been dedicated to uncovering the working mechanism of the Li–S system from experiments and theoretical calculations that greatly promote the development of lithium and sulfur electrodes. **Figure** **3** shows the schematic representation and the charge‐discharge profile of a typical Li–S battery. Nowadays, a widely accepted mechanism is as follows:

(1) $$S_{8}+2Li^{+}+2e^{-}↔Li_{2}S_{8}$$

(2) $$3Li_{2}S_{8}+2Li^{+}+2e^{-}↔4Li_{2}S_{6}$$

(3) $$2Li_{2}S_{6}+2Li^{+}+2e^{-}↔3Li_{2}S_{4}$$

(4) $$Li_{2}S_{4}+2Li^{+}+2e^{-}↔2Li_{2}S_{2}$$

(5) $$Li_{2}S_{2}+2Li^{+}+2e^{-}↔2Li_{2}S$$

In the discharging process, the lithium anode is oxidized to Li^+^ and releases an electron to the sulfur cathode via the external circuit. In the cathode, the reaction process is more complicated, which involves multielectron conversion electrochemistry from cyclo‐S_8_ to Li_2_S. Initially, the S_8_ is reduced to a series of long‐chain LiPS (Li_2_S*_n_*, 4 ≤ *n* ≤ 8) at 2.3 V, which corresponds to the first voltage plateau in the discharge profile with a theoretical specific capacity of 418 mAh g^−1^. These soluble LiPS are further reduced to solid Li_2_S_2_/Li_2_S at 2.1 V, which corresponds to the second voltage plateau in the discharge profile with a theoretical capacity of 1254 mAh g^−1^. For the subsequent charge process, Li_2_S is reconverted via polysulfides to S_8_, which is the reverse process of discharging.

### 2.2 Main Barriers of Li–S Batteries

Although the high specific capacity and energy density traits, several main barriers still exist and hamper the realistic application of Li–S batteries. The electrochemical chemistries and the barriers involved in Li–S batteries are summarized in Figure 3.

#### 2.2.1 For the Lithium Anode

Lithium possesses a high theoretical specific capacity of 3860 mAh g^−1^ and the lowest reduction potential (‐3.04 V vs standard hydrogen electrode), making it an ideal anode for an electrochemistry energy storage system.^[^ ^42^ ^]^ Nevertheless, the employ of lithium anode is the biggest challenge for the commercial application of Li–S batteries: 1) the lithium dendrites overgrowth caused by the inhomogeneous distribution of current density and Li^+^ concentration gradient will result in the direct electrical contact of cathode and anode and short circuit of a battery; 2) volume fluctuation during the Li^+^ plating/stripping process in the lithium anode will lead to the low utilization rate and the formation of dead Li; 3) high reactive lithium spontaneously reacts with organic electrolytes to form unstable SEI, which will result in continuous electrolyte and lithium consumption; 4) the surface passivation of lithium caused by the formation of Li_2_S from “shuttle effect” will increase the mass transfer in the cell.

#### 2.2.2 For the Sulfur Cathode

Element sulfur, featured as earth‐abundant, environmentally benign, and high specific capacity in Li–S batteries, is still suffer some notorious issues as follows: 1) the insulating nature of sulfur and Li_2_S severely limiting the electrochemical reactions and lead to the low utilization of active materials in Li–S batteries; 2) the large density difference of S_8_ and Li_2_S lead to a huge volume fluctuation during redox process, which will result in the collapse of the cathode structure; 3) high solubility of LiPSs in the organic electrolyte lead to the “shuttle effect” in Li–S batteries, severely declining the Coulombic efficiency, specific capacity, and cycle stability; 4) high concentration LiPSs in the condition of high sulfur loading state causes the sluggish kinetics in redox reactions, especially in the transformation process of Li_2_S_4_ to Li_2_S.^[^ ^43^ ^]^

#### 2.2.3 For the Separator

The separator, a fundamental and critical part of the Li–S battery system, generally is a porous polymer membrane and easy to transport lithium ions but electrical insulation.^[^ ^19^, ^44^ ^]^ Although the separator does not directly participate in the redox reaction in Li–S chemistry, its properties intensively affect the electrochemical performance of specific capacity, cycle life, and safety, which include wettability, chemical stability, mechanical strength, and porosity. Taking the notorious "shuttle effect" into consideration, the separator that serves in the Li–S battery system is required more rigorous demands to suppress the LiPSs shuttle. More importantly, the safety of the battery system is highly relevant to the quality of the separator, thus the stability and heat resistance of the separator need to be concerned.

## 3 Electrospinning Technique and the Nanofibers in Li–S Batteries

### 3.1 Parameters on Fibers Formation

**Figure 4:**
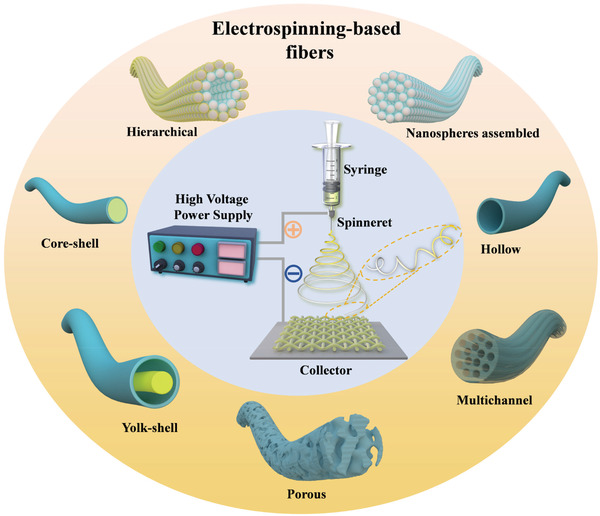
Schematic illustration of electrospinning technique and various electrospun nanofibers.

Electrospinning first invented as a novel patented technique to prepare superior nanofiber was in 1934,^[^ ^45^ ^]^ which is a fiber‐spinning procedure, using high static voltage to generate fibers with adjustable diameters ranging from a few nanometers to micrometers.^[^ ^46^ ^]^ The basic part of electrospinning includes a high‐voltage supply as a driving force, a syringe connects with a spinneret to generate a pendant droplet, and a conductive collector to collect fibers, as shown in the center circle of **Figure** **4**.

During the electrospinning process, a pendant droplet is first formed on the spinneret because of the liquid surface tension. Under the electrostatic field, the electrostatic repulsion from the surface of the pendant droplet deforms them into a Taylor cone, from which a charged jet is ejected when the electrostatic repulsion exceeds the surface tension. From the jet to fibers need a long‐distance change process, which includes a stable straight‐line segment and other three bending instabilities segments of vigorous whipping motions. Until the jet stretches to a finer diameter, the solvent evaporates and a solid fiber is formed, deposited on the conductive collector. In summary, the process for the formation of fibers by electrospinning could be divided into five steps: i) forming the Taylor cone under the electrostatic field; ii) reaching the critical voltage to form ejected jet; iii) stable straight‐line extension segment of the charged jet; iv) whipping instability of the jet in the electric field to thinning the jet; and v) solvent evaporation segment to form solid fibers and collection. Furthermore, the electrospinning process will be affected by solution parameters, process parameters, and environmental parameters, which will directly affect the quality of fibers. A detailed discussion of these parameters is listed in the following sections.^[^ ^47^ ^]^

#### 3.1.1 Solution Viscosity of Polymers

For polymers solution electrospinning, the solution viscosity is an important parameter that directly influences the quality of electrospinning‐based fibers even the spinnability. The solution with low viscosity will decline the stretching ability of the jet due to the weak entanglement between polymer chains, and the electrostatic force is dominant, forming mist particles or bead shape fibers. With the increase of viscosity, the entanglement between polymer chains will be enhanced, which is easier to form fibers with higher continuity and uniform diameter. When the solution with too high viscosity, viscoelastic resistance will play a dominant role in the electrospinning process, which is averse to form a jet, preventing the formation of fibers. Therefore, choosing a reasonable solution viscosity is very important for electrospinning.^[^ ^48^ ^]^

#### 3.1.2 Surface Tension and Electrical Conductivity

In the electrospinning process, surface tension and electrical conductivity are a pair of parameters that balance each other on the control of fibers formation. The electrostatic force must overcome the surface tension from the pendant droplet, then the electrospinning process could be continued. Therefore, high electrical conductivity is needed in the solution to guarantee sufficient electrostatic force. The electric field force tends to stretch the droplet and increase the jet surface area, while the surface tension tends to maintain the spherical surface of the droplet and reduce the jet surface area. Thus, choosing a proper conductivity and surface tension of solution has an important influence on the final morphology of fiber. Furthermore, the surface tension can be controlled by adjusting the solution viscosity, solvent composition or adding surfactants, which could reduce the critical voltage and regulate the fiber morphology. The conductivity of the solution can also be regulated by adding salt, polyelectrolyte, or highly conductive nanofillers, as well as adjusting the surface tension to regulate the fiber morphology and diameter.^[^ ^49^ ^]^

#### 3.1.3 Voltage

Voltage is a very important parameter, which directly affects the electric field force on the Taylor cone and jet in the electrospinning process. Therefore, a critical voltage is needed from sufficient electrostatic force to overcome the surface tension. In general, the high voltage will be better to accelerate jet stretching and obtain thin diameter fibers, but the too high voltage will puncture the jet which will affect the quality and collection of the fiber.

#### 3.1.4 Collection Distance of the Fibers

The collection distance is generally the distance from the spinneret to the conductive collector. Solution electrospinning requires a sufficient distance before the fiber is formed to allow the solvent to evaporate, thus, a minimum collection distance is required. It has been found that too long or too short collection distance will lead to the formation of beaded fibers, and the effect of collection distance on the morphology of fibers is different for different types of the polymer electrospinning process. For example, Lee et al.^[^ ^50^ ^]^ studied the spinning performance of PVC solution and found that when the collection distance increased from 10 to 15 cm, the diameter of the prepared PVC fiber decreased about 300 nm on average. This finding indicates the collection distance has a significant effect on fiber size.

#### 3.1.5 Flow Rate of the Solution

Flow rate is another parameter that cannot be ignored in the electrospinning process. Much high flow rate will eject too much solution which could not fully evaporate the solvent from fibers. In general, the high voltage could accelerate the jet stretching. Therefore, a high flow rate could couple with high voltage to speed up the evaporate rate of solvent to obtain higher fibers production rate. Furthermore, lower flow rates will result in the discontinuity of the solution, forming discontinuous fibers.

#### 3.1.6 Temperature and Humidity

Increasing the temperature could speed up the evaporation rate of solvent in the electrospinning process, but too high a temperature will lead to too quickly solvent evaporates on the spinneret, which could block the solution flow. Some studies have shown that humidity can increase fiber diameter by reducing the stretching effect, thus obtaining thick fibers, and higher humidity can increase pore formation. Furthermore, too low humidity will also expedite the evaporation rate of solvent, resulting in the blocked spinneret.

#### 3.1.7 Polymers

Polymers dissolve in the solvent making precursor solution is the prerequisite in electrospinning, thus, polymer occupies a special position in the electrospinning process. Many types of polymers could serve as a precursor in electrospinning, which is summarized in **Table** **1**.^[^ ^51^ ^]^ Importantly, the soluble metal source or functional groups can be added into the precursor solution, causing a diversity in the components, structures, and functions of electrospinning‐based fibers. It is precisely because of the diversity in structure and composition of electrospinning‐based nanofibers that could be targeted design in response to wide applications.^[^ ^9^, ^22^ ^]^ Recently, the improvements in spinning equipment such as needles and high‐speed collectors also provide more convenience in adjusting the nanofiber structure.^[^ ^45^, ^52^ ^]^

**Table 1:** Representative polymers and their corresponding electrospinning parameters

Polymer	Solvent	Concentration [%]	Voltage [kV]	Collect distance [cm]	Flow rate	Temperature and humidity	Refs.
PAN	DMF	9	15	10	0.6 mL h^−1^	/	^[^ ^32^ ^]^
PVP	Ethanol/acetic acid/water	5	15	15	1.5 mL h^−1^	25 ± 2 °C 45±5%	^[^ ^113^ ^]^
PI	NMP	15	+15, ‐1	15	10 µL min^−1^	/	^[^ ^30^ ^]^
PVA	Water	15	22	18	1.5 mL h^−1^	25 ± 2 °C 45 ± 5%	^[^ ^34^ ^]^
PEO	Water	8.4–12.5	15–20	17	/	20%	^[^ ^114^ ^]^
PMMA	DMF	12	12	15	4 mL h^−1^	25 °C 40%	^[^ ^115^ ^]^
PS	DMF	10–30	10–20	5–20	0.5–2 mL h^−1^	25 °C	^[^ ^116^ ^]^

### 3.2 Superiority of Using Electrospinning Technique in Li–S Batteries

**Figure 5:**
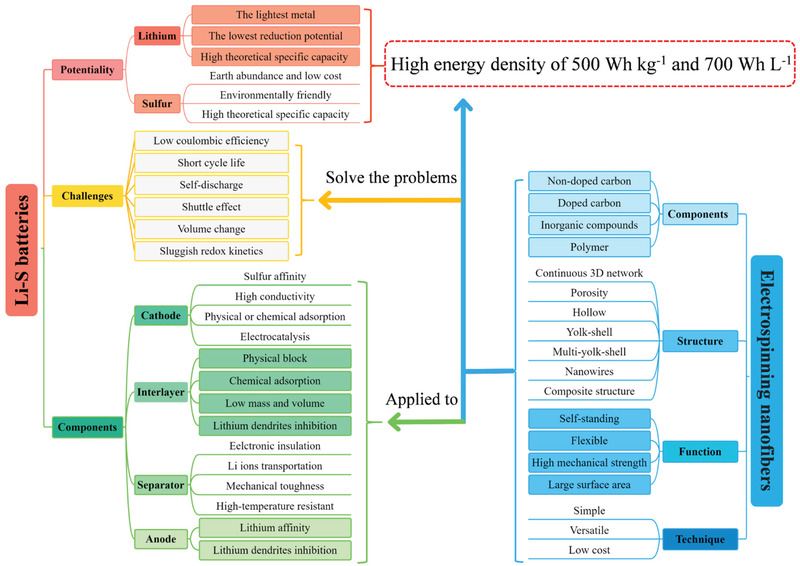
Schematic description of Li–S battery system and the electrospinning‐based nanofibers.

As shown in Figure 4, there is significant diversity in the composition and nano‐morphology of the electrospinning‐based nanofibers. The stabilization and heat treatment process of the electrospinning‐based nanofibers can also improve the component diversity. Under this condition, the single structure of nanofibers with diverse phases (polymer, carbon, metallic compound) and morphologies (hollow, porous, core‐shell, yolk‐shell) could be obtained.^[^ ^53^ ^]^ For a more systematical representation, a visualized summary of electrospinning‐based nanofibers as well as the Li–S battery system is present in **Figure** **5**, the merits and demerits of Li–S batteries and the functional requirements of each component are exhibited in the left part while the various design elements of electrospinning‐based nanofibers materials are shown in the right part. It is precisely because of the diversity in structure and composition of electrospinning‐based nanofibers that could be targeted design by researchers in response to the problems of Li–S batteries to achieve high *W*_G_ and *W*_V_ of 500 Wh kg^−1^ and 700 Wh L^−1^, respectively.

**Figure 6:**
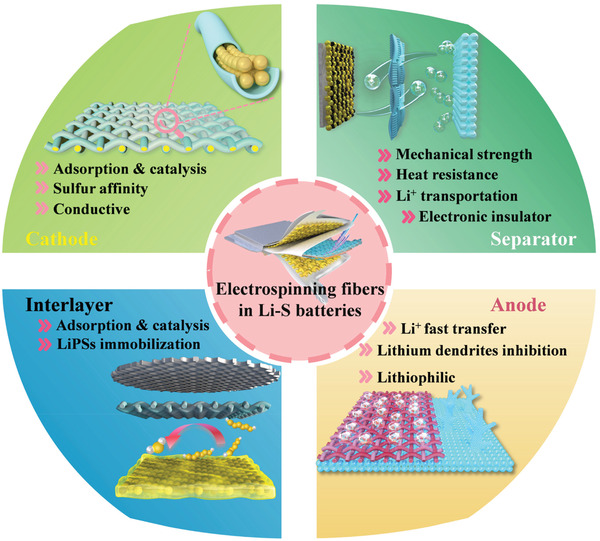
Schematic illustration of the electrospinning‐based nanofiber materials used in the four main parts of Li–S batteries.

As shown in **Figure** **6**, the electrospinning‐based nanofibers used in Li–S batteries could be divided into four categories of sulfur cathode host, interlayer, separator, and lithium anode host. For the cathode part, the porous and hollow structures of nanofibers show a large hollow space to accommodate a large mass of sulfur, and the conductive carbon‐based skeleton can enhance the conductivity of the sulfur cathode, thus improving the reaction kinetics in the Li–S system.^[^ ^4^, ^5^ ^]^ As for the interlayer part, the LiPS interception effect of a complete interwoven nanofiber membrane is also a novel and effective attempt.^[^ ^54^ ^]^ The isolated functional part of adsorption and catalytic would also advance the redox reaction efficiency in Li–S batteries.^[^ ^55^ ^]^ Besides, the electrospun polymer nanofibers have a good insulation nature, and the uniform holes between interwoven nanofiber networks could accommodate the rapid transfer of Li ions, making them promising candidates as the separator in Li–S batteries.^[^ ^19^, ^56^ ^]^ More importantly, the ingredient of nanofibers is easy to combine with some traditional heat‐resistant materials by electrospinning methods, achieving a significant improvement in the heat resistance of the separator.^[^ ^57^ ^]^ Recently, many published works reported that the nanofiber structural materials are ideal host candidates to accommodate lithium and restrict the dendrite overgrowth owing to their porous structure and high surface area.^[^ ^58^ ^]^ Some compounds that have been proved to have a good lithium affinity could also be added into the nanofiber skeleton, further improving the ability in Li dendrites inhibition.^[^ ^59^ ^]^ Except for the specialties above discussed, electrospinning‐based nanofibers possess outstanding flexibility and stretchability, making them unique components for flexible Li–S batteries.

With the increasing maturity of electrospinning technology and the rapid evolutions of nanotechnology for the past few years, the association of electrospinning technology and other novel nanotechnology also provides us with new methods in preparing nanofiber materials with diverse hierarchical architectures.^[^ ^60^ ^]^ The various nano methods as a hydrothermal method, hydrolysis method, and chemical vapor deposition (CVD) technique are easy to combine with the electrospinning technology in constructing multifunctional nanofiber‐based material with the enhanced specific performance or fantastic morphological characteristics.^[^ ^61^ ^]^ Different from other nanotechnology, electrospinning shows an irreplaceable advantage in macroscopic integrity due to the scale of the electrospinning membrane can easily reach up to the large size of 20 cm × 20 cm, showing a broad practical application prospect.^[^ ^53^ ^]^

## 4 Crucial Parameters of Dominating the *W*_G_ and *W*_V_ in Li–S Batteries

To achieve the high *W*_G_ and *W*_V_ of Li–S batteries in commercial applications based on the electrospinning‐based nanofibers, attention should be paid not only to the study of the material but also to the electrode structure and cell component engineering. Therefore, we emphasized that, from the fundamental research summaries, specific capacity, electrolyte/sulfur (E/S) ratio, sulfur loading, and cathode tap density are crucial parameters for achieving high *W*_G_ and *W*_V_ in Li–S batteries. Furthermore, we put forward a mathematical model based on the electrospinning‐based nanofibers serving as a cathode and anode host, and separator to probe the crucial parameters of dominating the *W*_G_ and *W*_V_ in Li–S batteries.^[^ ^62^ ^]^

### 4.1 The Influence of Specific Capacity and Sulfur Loading on *W*_G_ in Li–S Batteries

Although Li–S batteries are well known as high specific capacity of 1675 mAh g^−1^, they cannot be fully achieved at the practical conditions owing to the instinctive nature of sulfur and Li_2_S, especially with high sulfur loading in the cathode. Therefore, the batteries with high specific capacity are the precondition for high *W*_G_. Given the complexity of Li–S batteries, it is necessary to consider all parameters of *W*_G_, include density, sulfur loading, N/P ratio, and E/S ratio. To simplify the mathematic model based on the electrospinning technique, we establish a model consisting of electrospinning‐based fibers with lithium as the anode, electrospinning‐based fibers with sulfur as a cathode, and electrospinning‐based fibers as a separator to probe the crucial parameters of dominating the *W*_G_ and *W*_V_ in Li–S batteries. With this model, we propose that *W*_G_ and *W*_V_ can be derived by the equation of:

(6) $$E_{G}=\left(U\cdot C\right)/\left(\sum m_{i}\right)$$

in which *U* is the average cell voltage of 2.1 V for Li–S batteries, *C* is the areal capacity of the cathode (mAh cm^−2^), *m*_i_ is the mass per unit square of various cell components (mg cm^−2^) including a cathode, an anode, separator, and electrolyte.

(7) $$E_{V}=\left(U\cdot C\right)/\left(\sum d_{i}\right)$$

in which *U* is the average cell voltage of 2.1 V for Li–S batteries, *C* is the areal capacity of the cathode (mAh cm^−2^), *d*_i_ is the thickness of various battery components (µm) containing a cathode, an anode, and separator. It should be noted that all parameters in the equation are at ideal conditions: 1) no volume expansion of the batteries in the charge and discharge process; 2) electrolytes do not occupy extra volume and are accommodated in the pores in the electrode and separator; 3) do not concern about the side reactions in the batteries.

**Figure 7:**
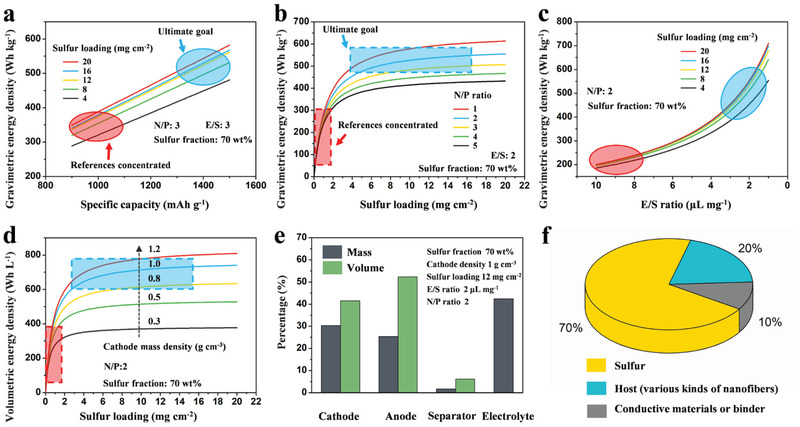
Key parameters of dominating *W*_G_ and *W*_V_ of Li–S battery: a) Dependence of *W*_G_ on specific capacity with different areal sulfur loading. b) Dependence of *W*_G_ on N/P ratio with different areal sulfur loading. c) Dependence of *W*_G_ on E/S ratio with different areal sulfur loading. d) Dependence of *W*_V_ on areal sulfur loading and cathode density. e) Volume and mass fraction of each component in full Li–S batteries. f) Schematic of a sulfur cathode component concluding of sulfur, host (various kinds of nanofibers), and conductive agent or binder. The area circled in red is where most of the references are concentrated, which means that the *W*_G_ and *W*_V_ of Li–S batteries from most reported works are less than 350 Wh kg^−1^and 400 Wh L^−1^, respectively. The area circled in blue is the ultimate goal of most researches, which is to achieve a high energy density of 500 Wh kg^−1^and 700 Wh L^−1^ for Li–S batteries.

Consideration of the importance of specific capacity with high sulfur loading in a battery, we first evaluate the influence of specific capacity at different sulfur loading for *W*_G_. As shown in **Figure** **7**a, the *W*_G_ has a linear relationship with specific capacity and tends to rise with the increase of sulfur loading. The *W*_G_ increases rapidly with the increase of sulfur loading less than 12 mg cm^−2^ but raising slowly when the sulfur loading is more than 12 mg cm^−2^. This phenomenon means that increasing the sulfur loading could not increase the *W*_G_ continuously. The calculated *W*_G_ is based on the assumptive parameters of N/P ratio = 3, E/S ratio = 3, and the sulfur fraction in the cathode is 70% in this equation. The correlation of *W*_G_ with N/P ratio at different sulfur loading is shown in Figure 7b. In ideal conditions, lithium and sulfur should possess the perfectly consistent capacity (N/P ratio = 1) in the batteries. However, the high reactivity of lithium to organic electrolytes will cause the irreversible consumption of lithium to form the unstable SEI, which means the N/P ratio is inevitably higher than 1. From the equation, we know that a high N/P ratio significantly decreases the *W*_G_. Therefore, the usage amount of lithium must be controlled in the battery and better bellowing 3 to guarantee the high *W*_G_. In this equation, the calculated *W*_G_ is based on the assumptive parameters of E/S ratio = 2, the specific capacity of 1200 mAh g^−1^, and the sulfur fraction in the cathode of 70%.

### 4.2 The Influence of E/S Ratio on *W*_G_ in Li–S Batteries

Except for sulfur loading, N/P ratio, and specific capacity, the E/S ratio is another crucial parameter for high *W*_G_ Li–S batteries. Excess injects of electrolytes not only give rise to some side reaction with lithium but also increase the mass of the batteries, resulting in the decrease of *W*_G_. Therefore, an assumption is established based on Equation (6) to evaluate the effect of the E/S ratio on *W*_G_, as shown in Figure 7c. In this assumption, the calculated *W*_G_ is based on the assumptive parameters of N/P ratio = 2, the specific capacity of 1200 mAh g^−1^, and the sulfur fraction in the cathode of 70%. The *W*_G_ decreases intensively with the electrolyte amount, meaning the inevitability of decreasing the E/S ratio in the Li–S batteries. Meanwhile, the same phenomenon could be found that *W*_G_ increases rapidly with the increase of sulfur loading less than 12 mg cm^−2^, but raising slowly when the sulfur loading is more than 12 mg cm^−2^. These assumptions reveal that high *W*_G_ could not be achieved only with high sulfur loading in the batteries and other parameters such as E/S ratio, N/P ratio must be optimized.

### 4.3 The Influence of Key Parameters on *W*_V_ in Li–S Batteries

*W*_G_ means the maximum energy supply amount at a limited mass in a battery, and *W*_V_ means the maximum energy supply amount at a limited space. Therefore, same as the *W*_G_, *W*_V_ is an important index to assess the electrochemical performance of a battery. To better determine the volume of the battery, the mass density of the cathode must be considered, and assume lithium anode has no pores. Here, mass density represents the ratio of the cathode mass to apparent volume, and can be calculated by Equation (8):

(8) $$\rho =\frac{m_{a}}{v_{e}}=\frac{\sum m_{i}}{v_{e}}=\frac{\sum w_{i}\rho_{i}}{v_{e}}\;$$

in which *m*_a_ represent the mass of the cathode (mg), concluding sulfur, host, conductive agent, or binder. *m_i_*, *w_i_* and *ρ*_*i*_ represent the mass (mg), the mass fraction (wt%), and true density (g cm^−3^) of the individual component of the cathode, respectively. *v_e_* represents the apparent volume of the cathode (cm^−3^). From this equation, we can deduce that the cathode volume is inversely proportional to the mass density, which means that high cathode mass density could enhance the *W*_V_ of a battery. As shown in Figure 7d, a model is established based on Equation (8) to evaluate the effect of cathode mass density on *W*_v_, which confirms the significance of cathode density in a battery and proves that the host or conductive agent should possess high mass density than sulfur. As shown in Figure 7e, the mass and volume percentage of cathode and electrolyte in a cell with a high energy density of 500 Wh kg^−1^ and 700 Wh L^−1^ taken the biggest part, suggesting their critical role in dominating the *W*_V_ and *W*_G_ in Li–S batteries.

**Table** **2** shows the key parameters to achieve an energy density higher than 500 Wh kg^−1^ and 700 Wh L^−1^, which includes an at least sulfur loading of 8 mg cm^−2^, a high cathode density of 1.2 mg cm^−3^, as well as the low E/S ratio of 2 and low N/P ratio of 2. More importantly, a higher energy density of 550 Wh kg^−1^ and 750 Wh L^−1^ could be achieved with the high sulfur loading of 12 mg cm^−2^ and high cathode density of 1.2 mg cm^−3^. Meanwhile, these results suggest that the sulfur loading is not the only parameter in controlling the energy density, which means that increasing the sulfur loading could not increase the *W*_G_ continuously. In conclusion, the electrospinning technique used in Li–S batteries could be an effective strategy to achieve high energy density batteries, and the cathode density and electrolyte dosage are the critical parameters in the *W*_V_ and *W*_G_, which must be concerned in the future Li–S batteries design.

**Table 2:** Representative energy densities at various parameters for Li–S battery

Sulfur loading [mg cm^−2^]	E/S ratio [µL mg^−1^]	N/P ratio	Cathode density [g cm^−3^]	*W*_G_ [Wh kg^−1^]	*W*_V_ [Wh L^−1^]
4	3	2	0.5	350	450
4	2	3	0.8	400	520
8	3	2	0.5	420	450
8	2	2	0.8	500	560
8	2	2	1.2	500	700
12	2	2	1.0	550	700
12	2	2	1.2	550	750

## 5 Recent Development on Electrospinning‐Based Nanofibers in Li–S Batteries

### 5.1 Cathode

As a crucial part of Li–S batteries, cathode, has been widely researched and reported in the past 10 years. With the great efforts of researchers all over the world, deeper understandings and more effective strategies for improving the conductivity of sulfur and Li_2_S have been achieved. For the nonconductive sulfur cathodes, the electrospinning‐based fibers with a 3D conductive network would be a suitable additive. The “shuttle effect” of polysulfides has been regarded as the most notorious problem for the sulfur cathodes, enormous work indicates the strong adsorption and efficient catalyst of polar additives to the intermediate polysulfides can effectively alleviate the “shuttle effect.” Through a modified electrospinning technology or a further modification of electrospinning‐based nanofiber, multifunctional fibrous nanohybrids with evenly distributed polar additives and catalytic nanoparticles could be fabricated. The 3D network structure of the fibrous nanohybrids could improve the contact between the polar or catalytic additive and the active sulfur, thereby improving the efficiency in adsorbing and catalyzing polysulfides.^[^ ^63^ ^]^ Besides, the nanofiber skeleton is tough enough in mechanical strength to maintain structural stability through the charge–discharge process, which is very essential in the long cycle life.

#### 5.1.1 Nondoped Carbon Nanofibers

Carbon nanofibers (CNFs) could be acquired by carbonization of the prepared electrospinning polymer nanofibers (Polyvinylpyrrolidone (PVP), polyacrylonitrile (PAN), Polyethylene Oxide (PEO), etc.). Compared with the previous 0D carbon support materials (carbon sphere, carbon black), the electrospinning CNFs have an ultra‐long 1D fiber structure and this continuous conduction path of the electron could reduce the contact resistance in the cathodes.^[^ ^64^ ^]^ With the improvement of electrospinning technology, the current electrospinning CNFs always have excellent mechanical properties, which is essential in sustaining the volume changes in the cathode during the charging–discharging process. Besides, the electrospinning CNFs interweave randomly into a 3D network architecture, which could effectively restrain the agglomeration of polysulfides or sulfur, thus increasing the utilization of active substances. In the early work of Zhang's research team, a porous CNF‐S nanocomposite cathode was fabricated based on electrospinning.^[^ ^23^ ^]^ The high conductivity and the surface area of the CNFs enabled the homogeneous dispersion and immobilization of sulfur species on the CNF substrates, delivering a high specific capacity of 1400 mAh g^−1^ at 0.05 C.

Besides, the crossed interwoven structure of CNFs has been proved to have advantages in depositing the active materials and trapped the viscous polysulfides by Lee's research team.^[^ ^32^ ^]^ In their work, a free‐standing CNFs membrane was fabricated by carbonization the electrospinning PAN under nitrogen. After being immersed in a sulfur‐containing slurry, which is easily loaded onto the CNFs matrix, indicating good compatibility. Profiting from the ultrahigh conductivity of the unique fiber and the excellent physical confinement of sulfur species, the freestanding CNFs cathode with a high sulfur loading of 10.5 mg cm^−2^ maintained high Coulombic efficiency and showed a high an excellent capacity retention rate of 90.3% in 100 cycles. Meanwhile, a high areal capacity of greater than 7 mAh cm^−2^ was realized, showing a practical prospect.

Normally, the electrospinning CNFs films are composed of numerous interwoven nanofibers along with considerable mesopores. However, the single nanofiber in CNFs films is composed of carbon materials, leading to a low specific surface area. The construction of hierarchical pore architecture in the nanofiber substrate has been regarded as an efficient way to improve the site capable of supporting polysulfide due to the increased surface area of the CNFs matrix. In recent years, many researchers have developed plenty of effective methodologies for preparing porous carbon nanofiber materials, including the template method, multiphase separation, and in‐situ introduction of nano‐hollow materials, which can easily ameliorate the carbon substrate of nanofibers and introduce hierarchical mesoporous.

**Figure 8:**
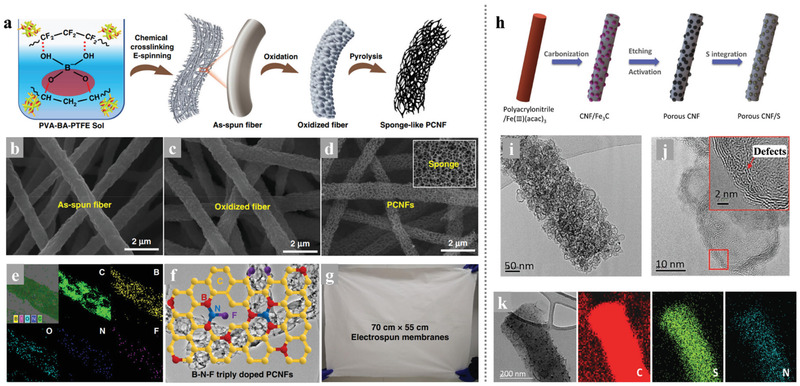
a) A general picture of using the chemical crosslinking electrospinning method to synthesize PCNFs. b–d) SEM images of the as‐spun fibers, the oxidized fibers, and the PCNFs, e) EDS mapping spectrum of PCNFs, f) The proposed chemical model of B–N–F doped PCNFs, g) A digital photo of the as‐spun film with a size of 70 cm × 55 cm. Reproduced with permission.^[^ ^34^ ^]^ Copyright 2019, Springer Nature. h) Schematic illustration of the synthesis of porous CNF/S composites: CNF/Fe_3_C containing graphitic carbon layer after carbonization; porous CNF after etching Fe_3_C and chemical activation; porous CNF/S by infiltrating sulfur. i,j) TEM and HRTEM images of PCNF/A550. k) TEM images and corresponding EDS elemental maps of PCNF/A550/S fiber. Reproduced with permission.^[^ ^66^ ^]^ Copyright 2017, Wiley‐VCH.

The CNFs with a porous structure not only have excellent conductivity but also enable fast access by lithium ions and polysulfide molecules, especially applicable for the Li–S battery system. Ding et al.^[^ ^34^ ^]^ developed novel nanotechnology that combines chemical crosslinking electrospinning and dual‐phase separation method as shown in **Figure** **8**a. After planned heat treatment in the N_2_ atmosphere, B‐, F‐, and N‐ atoms appeared in the PCNFs without any infiltrating dopants process except the inner reactions between N_2_, boric acid (BA), and poly (tetrafluoroethylene) (PTFE) during the high‐temperature pyrolysis. The porous CNFs (PCNFs) fabricated in this way show excellent conductivity, large pore volumes, high flexibility, and large‐scale integrity (Figure 8b–g). When served as scaffolds in sulfur cathodes, these PCNFs show excellent performance: high capacity was 1380 mAh g^−1^, and the discharge capacity decreased to 1000 mAh g^−1^ after 300 cycles (72.5% retention rate), suggesting that the unique structure in these PCNFs could be competent in maximizing both sulfur loading and optimal ion diffusion and electronic conduction.

Normally, the most popular synthesis route for electrospinning PCNFs is the introduction and etching of nano‐templates in the nanofibers.^[^ ^65^ ^]^ The size and content of the nanoparticles as templates are the main control points of the preparation process, which will affect both the specific surface area and pores distribution of the synthesized PCNFs. Kim's research team fabricated PCNFs with the catalytic Fe_3_C particles as templates and explored the functions and roles of the micropores and mesopores in ameliorating the stubborn problems in Li–S batteries as the volume expansion and “shuttle effect.”^[^ ^66^ ^]^ As shown in Figure 8h–k, the catalytic effect of the Fe_3_C particles facilitates the generation of graphitic carbon layers in the low‐temperature procedure and leaving numerous pores in the carbon nanofiber matrix when the etch of Fe_3_C. Through the advanced in‐situ TEM measurement in the lithiation process of the cathode found that the PCNFs/S cathodes with different porous structures show significant differences in volume expansion. This phenomenon indicates that the pore size distribution of the porous structure in PCNFs/S cathodes would influence the ability to prevent the lithiation products from overflowing. As a result, the optimal PCNF/A550 with large pore volume and abundant micropores delivered a high capacity of 945 mAh g^−1^ at 1 C with outstanding cycle stability and high‐rate performance.

For the preparation of 0 D hollow spheres inside of electrospinning CNFs, selecting templates with spherical morphology which were in situ produced from the precursors inside the electrospinning‐based nanofibers during calcination would be a feasible choice. For instance, Wu et al.^[^ ^28^ ^]^ developed a novel method that using the Ni nanospheres as hard templates, which were transformed from the in situ introduction of Ni(Ac)_2_, to synthesize PCNFs with hollow graphitized carbon spheres after removing the catalytic Ni nanospheres. Besides, directly adding spherical nanoparticles into the electrospinning solution would also be an approach. As the most popular sacrificial template, the SiO_2_ nanosphere could be introduced into the electrospinning CNFs and serve as a porogen to produce PCNFs. Combining the SiO_2_ template sacrifice and KOH etching activation with the electrospinning technology, Yang et al.^[^ ^67^ ^]^ synthesized flexible 3D porous CNFs with hierarchical porous structures. After a melt‐diffusion process, sulfur can easily immerse into the pores of the PCNFs and construct a binder‐free cathode. The 3D interconnected conductive CNFs network in the cathode provides multiple transport paths for electrons and Li^+^, and the hierarchically porous structure provides larger specific surface and pore volume as well as the essential function in alternatively reserving the polysulfide. As a result, this composite electrode displayed an outstanding discharge capacity of 1422.6 mAh g^−1^ at 0.2 C with a high sulfur content of 76 wt%, an excellent energy density of 754 Wh kg^−1^, and a power density of 1901 Wh kg^−1^, which greatly exceeded the energy/power density of previous reported Li–S batteries.

**Figure 9:**
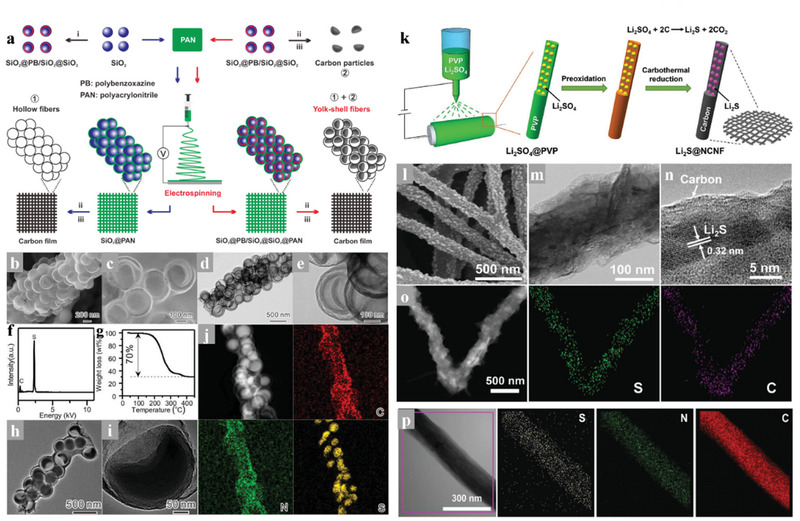
a) Schematic illustration of the templated electrospinning strategy for the fabrication of the yolk‐shell carbon fiber network, b,c) SEM images of the BCN@HCS fibers, d,e) TEM images of the BCN@HCS fibers, f) EDX spectrum, g) TGA curve, h,i) TEM images, and j) STEM image and corresponding elemental mapping. Reproduced with permission.^[^ ^68^ ^]^ Copyright 2018, Elsevier. k) Schematic illustration of the production of freestanding flexible Li_2_S@NCNF paper electrodes via Ar‐protected carbothermal reduction of Li_2_SO_4_@PVP fabrics made by electrospinning at ambient conditions. l) FESEM image of Li_2_S@NCNF paper, m) TEM image of a Li_2_S@NCNF fiber, n) HRTEM image showing the decoration of single‐crystalline Li_2_S nanoparticles in carbon nanofibers with disordered structure, o) elemental mapping visualizing the uniform distribution of sulfur and carbon elements in Li_2_S@NCNF, p) TEM image and corresponding elemental mapping showing the uniform distribution of sulfur, nitrogen, and carbon elements in Li_2_S@NCNF electrode after 200 cycles. Reproduced with permission.^[^ ^71^ ^]^ Copyright 2017, Wiley‐VCH.

In the designing and construction of PCNFs for the cathodes of the Li–S battery system, we should take into great consideration that the size of the pores around the CNFs outer wall should be minimized to avoid outer diffusion of polysulfides. However, it is still technically immature to achieve uniformly selective hierarchical porous structure on a macro‐scale self‐standing CNFs membrane. Instead, introducing the nano‐hollow materials with inherent shells into the carbon substrate could efficiently construct closed hollow space in the CNFs. Lin et al.^[^ ^68^ ^]^ developed a simple electrospinning technique for fabricating the interconnected yolk–shell carbon nanospheres assembled fiber network to prepare self‐supported sulfur cathodes as shown in **Figure** **9**a–j. The multi yolk‐shell nanospheres‐assembled CNFs not only inherited the advantages of the yolk‐shell structure which could achieve perfect encapsulation of active sulfur and polysulfides but also maintained the high conductive network and self‐standing skeleton of electrospinning CNFs membrane. With the particular yolk‐shell structure and interconnected conductive networks, the free‐standing yolk–shell CNFs cathode with a high sulfur content of 70 wt% and loading 4 mg cm^−2^ achieved a high discharge capacity of 1083 mAh g^−1^, excellent rate capacity of 562 mAh g^−1^ at 4 C, and outstanding cycle stability. Moreover, a high areal sulfur loading of 16 mg cm^−2^ was achieved and exhibited a superior area capacity of 15.5 mAh cm^−2^ with a high sulfur utilization of 57.8%.

Recently, the full lithiation phase of sulfur, Li_2_S, has been used as active materials in Li–S batteries for its high capacity of 1166 mAh g^−1^ and unique structural stability.^[^ ^69^ ^]^ The Li_2_S phase occupies the maximum volume in the multi‐step electrochemical reaction in the Li–S battery system, thus fundamentally avoiding the structural damage caused by volume expansion. Besides, the safety issues induced by the lithium dendritic growth can be greatly solved since the lithium‐free anodes as the applied cathode of Li_2_S. In 2014, Cui et al. performed pioneering work that successfully encapsulated Li_2_S into the 2D layered titanium disulfide that offered high conductivity and affinity for polysulfides.^[^ ^70^ ^]^ The as‐prepared nanocomposite cathodes exhibited enhanced cycle stability in Li–S batteries, indicating the practicality of this encapsulation strategy. In addition to the layered 2D structure, the hierarchical carbon structure could also be competent in encapsulating Li_2_S. Considering the high conductivity and the self‐standing characteristic of electrospinning CNFs, encapsulating Li_2_S into the carbon matrix of CNFs seems to be an intriguing strategy. Qiu's research team has tried this strategy and achieved it in synthesizing freestanding flexible Li_2_S‐based paper electrodes with ultrahigh Li_2_S loading.^[^ ^71^ ^]^ As shown in Figure 9k, the Li_2_S/NCNF was fabricated through easy carbonization of the electrospinning polyvinyl pyrrolidone (PVP)/Li_2_SO_4_ precursor, in which the PVP served as the carbon source and Li_2_SO_4_ was in situ reduced into the active material source (Li_2_S) by the carbon matrix. The unique structure with ultrafine Li_2_S nanoparticles encapsulated in the CNFs shows a good adhesion between the Li_2_S and the carbon substrate. This gives rise to a mighty electrochemical integrated circuit, leading to the spatial separation of active material particles but electrically interconnected, which could enhance the electrode uniformity with less compromise of the conductivity in CNFs (Figure 9l–p). Therefore, this free‐standing Li_2_S@NCNF cathode at a Li_2_S load of 3.0 mg cm^−2^ shows a high capacity of 730 to 460 mAh g^−1^ with a rising current rate of 0.2 to 2.0 C as well as durable cycling performance. Moreover, such a free‐standing Li_2_S@NCNF cathode could be stacked layer by layer to realize a high Li_2_S loading of 9.0 mg cm^−2^ with a high areal capacity of 5.76 mAh cm^−2^, and the stacked cathode still shows good capacity retention even at a high current density of 1.0 C.

#### 5.1.2 Composite Carbon Nanofibers

Although the structural design of electrospinning carbon nanofibers has improved the physically blocking ability of LiPSs, the physical obstruction of LiPSs is not effective to restrict the shuttle effect owing to the nonpolar surface of the unmodified carbon material shows poor adsorption and catalytic capabilities for polysulfides.^[^ ^72^ ^]^ Chemical adsorption to LiPSs from some transition metal could effectively suppress the shuttle effect owing to the strong chemical bond in‐between, which could further facilitate the uniform distribution of sulfur species on the carbonaceous materials thereby enabling strong electrical contact in the cathode. Catalysts such as oxides, nitrides, sulfides, and heterostructures atoms could enhance the redox reaction kinetics and decrease the accumulation amount of LiPSs on the surface of cathodes, which is beneficial to mitigating the shuttle effect. Fortunately, electrospinning is a diversified technology that could be feasible to modify the precursor solution as the addition of metal ions and compounds, thus introducing appropriate adsorbing and catalytic additives. Moreover, the metal ion and compound additives introduced into the electrospinning‐based nanofibers will undergo a phase change and redistribution during the subsequent heat treatments, which might form a marvelous nanostructure and promote the main function as adsorption and catalytic of the polar additives in the Li–S batteries.

**Figure 10:**
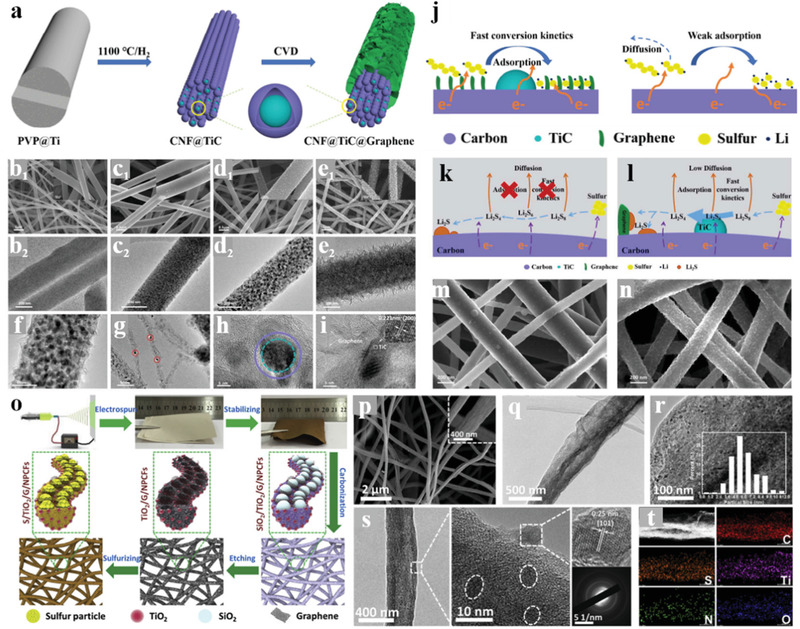
a) Schematic illustration of the fabrication process for multi‐yolk/shell structured CFTG‐1 composite. SEM and TEM images of the obtained b) CF, c) CFT, d,f–i) CFTG‐1 and e) CFTG‐2 composite. j) Schematic illustration of the Li_2_S nucleation and growth on CFTG‐1composite surface (left) and on carbon surface (right). Schematic illustration of LiPS redox reaction and Li_2_S nucleation on the k) CF and l) CFTG‐1 substrates. SEM images of m) CF/S and n) CFTG‐1/S cathodes at the discharged states with a sulfur loading of 1.2 mg cm^−2^ at 0.1 C. Reproduced with permission.^[^ ^74^ ^]^ Copyright 2020, Elsevier. o) Schematic illustration of the fabrication process for the S/TiO_2_/G/NPCFs composite. p) SEM and q, r) TEM images of TiO_2_/G/NPCFs (inset is the column diagram of the TiO_2_ particles' size distribution). s) TEM and SAED images of S/TiO_2_/G/NPCFs. t) Elemental mapping of S/TiO_2_/G/NPCFs. Reproduced with permission.^[^ ^75^ ^]^ Copyright 2017, Elsevier.

Many transition metal oxides (such as VO_2_, MnO_2_, TiO_2_, etc.) have been adopted in the Li–S batteries owing to the strong adsorption ability to LiPSs, which could suppress the shuttle effect.^[^ ^73^ ^]^ Nevertheless, the poor conductivity of the transition metal oxides limits their electrochemical performance in the sulfur cathode due to the inherent sluggish reaction kinetics of the Li–S batteries. Therefore, compounding with the highly conductive electrospinning CNFs would be a promising strategy in facilitating the application of the non‐conductive but adsorption strong polar materials in the cathode of Li–S batteries. Our groups have taken some efforts to fabricate electrospinning CNFs with modified nanostructure and components for further improvement in the cathodes of Li–S batteries. Zhang et al.^[^ ^74^ ^]^ reported a novel carbonaceous hybrid composite with vertical graphene grown on multi‐yolk/shell structured TiC@C nanofibers serve as sulfur host, enabling better electrochemical performance. The amazing structure of this nanofiber‐based composite is shown in **Figure** **10**a–n, it integrated synergistic characters of adequate volume, better adsorption, high active electrocatalysis, and high conductivity, thus delivered outstanding cycle stability even at a high sulfur loading. Song et al.^[^ ^75^ ^]^ spun the TEOS, TTIP, and GO into the PAN nanofibers, and prepared unique flexible NPCFs mixed with ultrafine polar TiO_2_ nanoparticles after carbonization and template etching. This TiO_2_/G/NPCFs film with excellent mechanical strength could serve as a free‐standing sulfur host in flexible Li–S cells. As shown in Figure 10o–t, the delicate construction of the TiO_2_/G/NPCFs composite not only integrated the outstanding conductivity of a carbon matrix and the strong chemical absorption ability of polar metal oxides but also well kept the flexibility of the film. After sulfuring, the fabricated S/TiO_2_/G/NPCFs cathode delivered a strongly enhanced performance.

**Figure 11:**
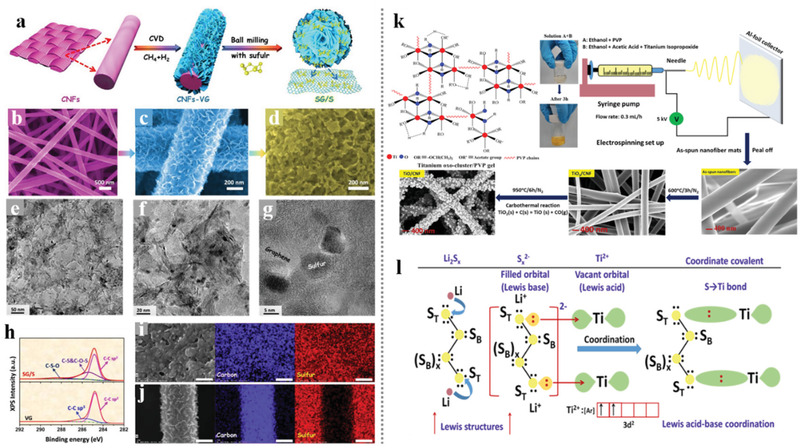
a) Schematic diagram showing the process for the fabrication SG/S composites and SEM images for b) CNFs, c) VG on CNFs, and d) SG/S. e,f) TEM and g) HRTEM images of SG/S. h) XPS C 1s spectra of VG on CNFs and SG/S. i) EDX mapping of the SG/S composite after 155 °C for 12 h. j) EDX mapping of the mixture of VG on CNFs and sulfur after 155 °C for 12 h. Scale bars: 200 nm for i,j). Reproduced with permission.^[^ ^76^ ^]^ Copyright 2020, Elsevier. k) Synthesis strategy for developing freestanding TiO/CNF nanofiber mats. l) A schematic explaining the Ti—S bond formation through coordination between unsaturated Ti‐centers (Lewis acid) and terminal S (S_T_) of S*_x_* ^2−^ (polysulfides). Reproduced with permission.^[^ ^77^ ^]^ Copyright 2018, American Chemical Society.

Compared with introducing graphene through the heat reduction of the graphene oxide spun into the nanofibers, the CVD methods directly using CNFs film as substrate could reach the hierarchical architectures of high‐quality graphene growing vertically on the CNFs matrix. Although high‐quality graphene is an ideal conductor for both ion and electron transport, the lack of defect limits its sulfur affinity and LiPS adsorption capacity. To achieve the tight bond between sulfur and the high‐quality vertical graphene‐modified CNFs and improving the adsorption of LiPSs, our group attempted much and presented an effective method in incorporating a large quantity of sulfur with vertical graphene flakes based on the ball‐milling technology.^[^ ^76^ ^]^ As shown in **Figure** **11**a–j, the sulfur could be combined well with the vertical graphene even at the atomic level, which is benefitted from the alloying effect during the ball milling procedure. Besides, the carbon‐sulfur bonds formed from the mechanical alloying effect could also endow the composite with an extra function of LiPS anchoring based on chemical adsorption. In the prepared ball‐milled CNFs/G/S cathode, the weakness of the non‐polar carbon‐based sulfur host in anchoring LiPSs was compensated by the excellent absorption ability of the introduced carbon‐sulfur bonds, thereby exhibiting an improving cycle and rate performance of the battery. Singh et al.^[^ ^77^ ^]^ reported a free‐standing TiO/CNF‐S cathode and proposed novel chemical anchoring effects to LiPSs through strong Lewis acid‐base interactions between terminal sulfur (S_T_) of lithium polysulfides (S*_x_* ^2−^) and unsaturated Ti‐centers in TiO nanofibers. Figure 11k,l shows the synthesis of the freestanding TiO/CNF nanofiber mats and the Ti—S bond formation based on the Lewis acid interactions. This work pioneered the chemical interaction between Lewis acid and base in the sulfur cathodes: the LiPSs can simultaneously possess polar‐polar (Li^+^‐O‐Ti) and Lewis acid‐base (S_Terminal_‐Ti‐O)‐type interactions with Ti‐O bonds. The incorporation of a strong 3D structure, high electrical conductivity, large surface area, and unique robust Lewis acid‐base interactions of TiO with LiPSs led to a high reversible discharge capacity and strongly enhanced cycle stability for TiO/CNF‐S cathodes with high sulfur loading.

**Figure 12:**
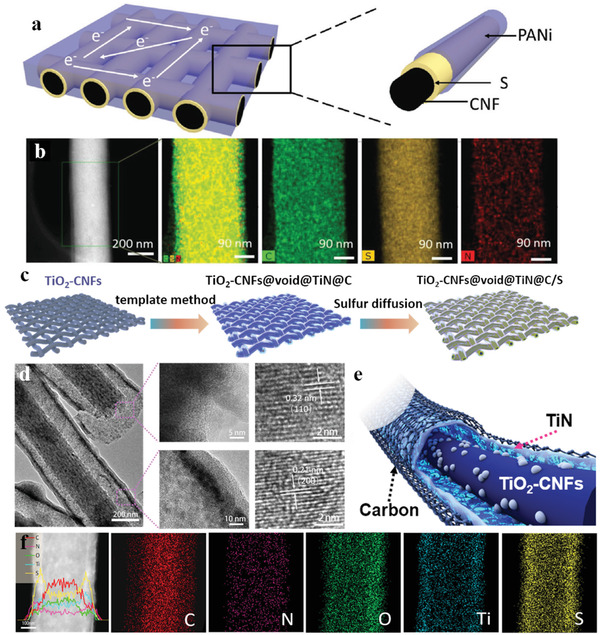
a) Schematic illustration of the CNF/S/PANi electrode configuration. b) STEM image of single CNF/S/PANi and corresponding elemental mappings of C, S, and N elements revealing the uniform distribution of S and PANi. Reproduced with permission.^[^ ^79^ ^]^ Copyright 2018, Wiley‐VCH. c) Schematic illustration of the synthesis process of TiO_2_‐CNFs@void@TiN@C/S cathode. d) TEM and HRTEM images of TiO_2_‐CNFs@void@TiN@C. e) Schematic illustration of the coaxial multilayered hollow structure of TiO_2_–CNFs@void@TiN@C. f) TEM image, linear elemental distributions, and area elemental distributions for C, N, O, Ti and S, of TiO_2_‐CNFs@void@TiN@C/S. Reproduced with permission.^[^ ^80^ ^]^ Copyright 2020, Elsevier.

The polar metallic compound doped into the electrospinning‐based nanofibers not only effectively prompts the chemical anchoring to the dissociative LiPS, but also provides novel ideas in facilitating the structure design of the nanofiber‐based composites. Li et al.^[^ ^16d^ ^]^ reported a marvelous nanocomposite with hollow TiO nanospheres packed into the electrospinning carbon nanofibers and constructed into a “grape cluster” structure. The compactly arranged TiO nanospheres played an important role in sustaining sufficient sulfur and limiting the loss of LiPS into the organic electrolyte, while the carbon skeleton played the roles of connection and conduction. The exquisite structural design enabled a high specific capacity of sulfur at various current densities even with a high content of 73 wt% in the GC‐TiO@CHF/S composite cathode. Meanwhile, superior cycling stability over 400 cycles could also be achieved in the high sulfur loading of 5.0 mg cm^−2^. Recently, Zhou et al.^[^ ^78^ ^]^ reported a similar template‐engaged electrospinning strategy and constructed 3D conductive network composite (CNB‐TiC@CNF) with a novel “boxes in fibers” structure. The cubic Fe_2_O_3_ nanocubes served as a template and played a key role in building isolated hollow carbon nano boxes along the nanofibers. Combined with the inherent high conductive 3D network, the ultra‐high sulfur loading capacity of the hollow carbon nano boxes, and the adsorption and catalytic function of the metallic TiC nanoparticles, this CNB‐TiC@CNF electrode revealed an outstanding performance for the practical implementation. Particularly, the CNB‐TiC@CNF cathode achieved a long cycle of 400 cycles with a decay rate of 0.15% even at a marvelous 10 C rate. Through a series of subsequent processing of the electrospinning‐based nanofibers, researchers have discovered a reliable sulfur encapsulation strategy based on the through space along the carbon nanofibers. For example, Zhu et al.^[^ ^79^ ^]^ dropped the aniline monomer onto a typical CNF/S cathode and polymerized it in situ to realize a full‐scale coating of the active sulfur. **Figure** **12**a,b shows the configuration schematic and nanostructure of this prepared (PANi)/S/CNFs (CNF) electrode, respectively. Compared with the traditional CNF/S cathodes, this effective 3D electrode fabricated in a simple process not only offered strong chemical adsorption and physical intercept effects of the LiPS, but also greatly improved the structural strength of the cathode to accommodate the volume fluctuation during cycling. Moreover, our research team also attempts a lot in constructing a continuous long‐ranged inner hollow space in the nanofibers. Zhang et al.^[^ ^80^ ^]^ introduced a freestanding carbon nanofibers film with a yolk–shell structure of a complete hollow yolk‐shell TiO_2_‐CNFs@void@TiN@C composite. As shown in Figure 12c–f, this multifunctional 3D structural design possesses the following merits: i) The double conductive carbon skeleton offered the enhanced electron transfer efficiency between the composite and sulfur; ii) The large hollow space could load much sulfur and sustain the volume expansion; iii) The polar TiO_2_ and TiN in the composite provided strong adsorption sites for the LiPSs; iv) The yolk–shell nanofiber structure effectively realized encapsulation of the LiPS.

#### 5.1.3 Noncarbonaceous Nanofibers

For the electrospinning‐based nanofibers used as sulfur hosts in the cathode of Li–S batteries, carbon is the most feasible and popular material choice of the nanofibers for its low density and high conductivity. Nevertheless, the carbon material is undoubtedly not the only choice in this field and many researchers have explored the usage of noncarbonaceous nanofibers matrix as hosts of sulfur, highly expanding horizons of material construction and theoretical exploration.

**Figure 13:**
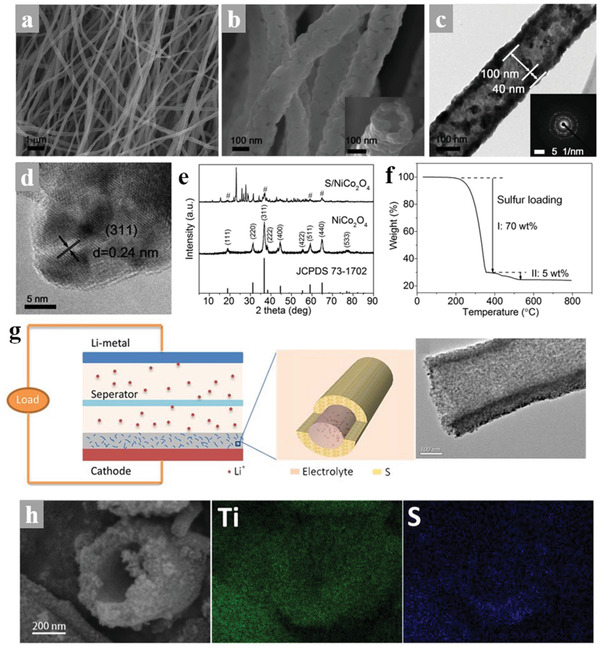
a,b) SEM images of NiCo_2_O_4_ nanofibers. c) TEM image of NiCo_2_O_4_ nanofibers and the corresponding SAED pattern. d) HRTEM image of NiCo_2_O_4_ nanofibers. e) XRD patterns of NiCo_2_O_4_ nanofibers and S/NiCo_2_O_4_ composite. The diffraction peaks of NiCo_2_O_4_ are marked with pound signs in the pattern of S/NiCo_2_O_4_ composite. f) TG curve of the S/NiCo_2_O_4_ composite in Ar atmosphere. Reproduced with permission.^[^ ^81^ ^]^ Copyright 2019, Wiley‐VCH. g) Schematic representation for high‐rate performance of MTDNTs/S composites cathode. h) STEM image of MTDNTs/S composite and elemental mapping of Ti and S. Reproduced with permission.^[^ ^82^ ^]^ Copyright 2017, Elsevier.

Currently, the polar metal compounds used as the LiPS adsorbents and catalysts in Li–S batteries are always combined with carbon materials for better functionalization but are unfavorable for the high *W*_V_ of the sulfur cathodes. Thus, the polar transition metal oxides were liberated from the carbon substrate and explored for a further enhancement in the tap density and *W*_V_ of Li–S batteries. As typical bimetallic‐based compounds, the spinel nickel cobaltite (NiCo_2_O_4_) with high density shows multiple catalytic active sites, which could intensively enhance the conversion kinetics of LiPS. Liu et al. reported a carbon‐free porous hollow 1D NiCo_2_O_4_ nanofibers composite as a sulfur host and fabricated the S/NiCo_2_O_4_ cathode with high sulfur content as shown in **Figure** **13**a–f.^[^ ^81^ ^]^ The NiCo_2_O_4_ nanofibers could achieve strong adsorption for LiPSs and provide strong active reaction sites to enhance their conversion process, even the tap density of the S/NiCo_2_O_4_ cathode reached a marvelous high level of 1.66 g cm^−3^, which is close to the theoretical density (2.07 g cm^−3^) of sulfur. Therefore, the S/NiCo_2_O_4_ composite delivered excellent volumetric capacity and cycle performance. Shen et al.^[^ ^82^ ^]^ reported mesoporous TiO_2_ nanotubes served as sulfur hosts in Li–S batteries, which were synthesized by electrospinning coupled with pyrolysis, enabling a high‐rate performance of 610 mAh g^−1^ at 8C and stable long‐stable cycling, as shown in Figure 13g,h. The author adopted PVP and tetrabutyl titanate as a precursor for electrospinning and further heated up to 550 °C in the air to prepare a hollow TiO_2_ nanotube serving as a sulfur host. The procedure is simple and effective and the structure is favorable for the fast transformation of Li^+^, but the lack of conductivity of TiO_2_ limits its application in the cathode with high sulfur content and loading. Furthermore, **Table** **3** shows the recent advance in the development of electrospinning‐based nanofibers materials for the cathodes of Li–S batteries.

**Table 3:** Electrospinning‐based nanofiber materials for the cathodes of Li–S batteries

Components	Structure	Electrochemical performance			Refs.
		Long cycle performance	Rate performance	With high sulfur loading	
Carbon (PAN), TiO	Hollow sphere cluster	/	620 mAh g^−1^ at 2 C	15 mg cm^−2^: 680 mAh g^−1^ at 0.2 C after 400 cycles	^[^ ^16d^ ^]^
Carbon (PAN)	Normal	/	/	10.5 mg cm^−2^: 680 mAh g^−1^ at 0.1 C (90.4% capacity retention)	^[^ ^32^ ^]^
Carbon	Porous	509 mAh g^−1^ at 1.5 C after 500 cycles (66.45% capacity retention)	553 mAh g^−1^ at 4 C after 200 cycles	/	^[^ ^66^ ^]^
Carbon (PAN)	Porous	/	688.4 mAh g^−1^ at 2C	/	^[^ ^67^ ^]^
Carbon (PAN‐PB)	Yolk–shell carbon nanospheres	700 mAh g^−1^ at 1 C after 500 cycles (82.8% capacity retention)	562 mAh g^−1^ at 4C	16 mg cm^−2^:15.5 mAh cm^−2^ at 0.1 C after 100 cycles	^[^ ^68^ ^]^
Carbon (PVP)	Li_2_S encapsulated	/	460–730 mAh g^−1^ at varied current rate of 0.2–2.0 C	Li_2_S loading of 9.0 mg cm^−2^: 520 mAh g^−1^ at 1C after 200 cycles	^[^ ^71^ ^]^
Carbon (PVP), TiC, graphene	Multi‐yolk/shell	620 mAh g^−1^ at 1 C after 800 cycles (68% capacity retention)	970.6 mAh g^−1^ at 2C	6.8 mAh cm^2^ with sulfur loading of 10.5 mg cm^2^	^[^ ^74^ ^]^
Carbon (PAN), TiO_2_, graphene	Porous	618 mAh g^−1^ at 1 C after 500 cycles (62.6% capacity retention)	668 mAh g^−1^ at 5C	4.8 mg cm^−2^: 490 mAh g^−1^ at 1 C after 100 cycles	^[^ ^75^ ^]^
Carbon (PAN), CVD graphene	Porous mixture	666 mAh g^−1^ at 0.5 C after 300 cycles (82.8% capacity retention)	506.5 mAh g^−1^ at 5 C	15 mg cm^−2^: 13.13 mAh cm^−2^	^[^ ^76^ ^]^
Carbon (PVP), TiO	Porous	518 mAh g^−1^ at 0.5 C after 200 cycles (65.5% capacity retention)	/	/	^[^ ^77^ ^]^
Carbon (PAN), TiC	Boxes in fibers	431 mAh g^−1^ at 10 C after 400 cycles (39.7% capacity retention)	739 mAh g^−1^ at 10 C	9.2 mg cm^−2^: 7.90 mAh cm^−2^ after 50 cycles	^[^ ^78^ ^]^
Carbon (PAN), PANi	PANi layer coating	552 mAh g^−1^ at 1 C after 300 cycles (71% capacity retention)	486 mAh g^−1^ at 2 C	2 mg cm‐2: 711 mAh g^−1^ at 0.2 C after 300 cycles	^[^ ^79^ ^]^
Carbon (PVP), TiO_2_, TiN	Yolk‐shell nanofiber structure	675.8 mAh g^−1^ after 1000 cycles at 1 C (68% capacity retention)	688.5 mAh g^−1^ at 5 C	9.5mg cm^−2^: 668.5 mAh g^−1^ after 300 cycles at 1.5 mA cm^−2^	^[^ ^80^ ^]^
SPAN, CNTs	Nanofiber@CNTs	1180 mAh g^−1^ after 800 cycles at 0.8 A g^−1^ (nearly 100% capacity retention)	885 mAh g^−1^ at 1.6 A g^−1^	4.0 mg cm^−2^: 1100 mAh g^−1^ at 0.8 A g^−1^	^[^ ^83^ ^]^
NiCo_2_O_4_	Hollow nanotube	487 mAh cm^−2^ after 1500 cycles at 1 C (41.5% capacity retention)	400 mAh g^−1^ at 5 C	4 mg cm^−2^: 834 mAh g^−1^ at 0.1 C	^[^ ^81^ ^]^

#### 5.1.4 Organosulfur Polymer Cathode Materials

**Figure 14:**
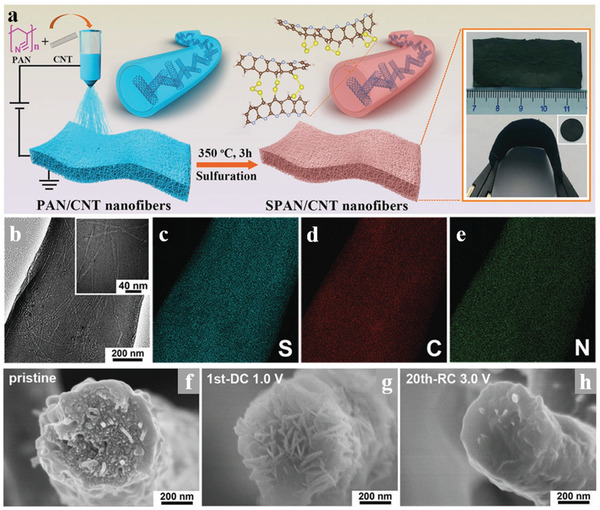
a) Schematic illustration for the synthetic procedure of SPAN/CNT electrodes. The inset orange boxes are digital pictures of the flexible and freestanding electrodes. b) TEM images of a single SPAN/CNT‐12 fiber and c–e) the corresponding elemental mapping of S, C, and N. The inset white boxes are images with high magnifications. f–h) Cross‐sectional SEM images of a single SPAN/CNT‐12 nanofiber at different discharge‐charge states. Reproduced with permission.^[^ ^83^ ^]^ Copyright 2019, Wiley‐VCH.

To suppress the shuttle effect of LiPS in Li–S batteries, many strategies have been adopted. Among them, the organosulfur polymer is a promising material due to the regulated structures and functionalities which could enhance the electrochemical performance of Li–S batteries. As a representative organosulfur polymer, sulfurized polyacrylonitrile (SPAN) could effectively restrain the shuttle effect by a solid–solid transformation upon the redox process in Li–S batteries. Combine with a simple synthesized process, SPAN has been widely used in the Li–S system. Wang et al.^[^ ^83^ ^]^ demonstrated a stable Li–S battery with flexible and freestanding SPAN as cathodes, as shown in **Figure** **14**, and the SPAN cathodes were constituted of a conductive 3D structure of electrospinning SPAN nanofibers. The structure and component characterization proved that the short sulfur chains were perfectly embedded in the *π*‐conjugated pyrolyzed PAN backbone, while the corresponding electrochemical characterization demonstrated the lithiation activity of sulfur even in a LiPS free process. The electrochemical reduction of SPAN by Li^+^ was a single‐phase solid‐solid reaction with Li_2_S as the sole sulfide product and the extra parasitic reaction between Li^+^ and C—N bonds make the backbone more conductive. The above characteristics enabled the SPAN/CNT cathode excellent rate performance and incredible cycle stability.

The interwoven framework of SPAN/CNT composite derived from electrospinning offers a freestanding and flexible fibrous cathode in Li–S batteries, which not only possesses fast ionic transport channels but also provides more space for Li_2_S deposition, greatly enhanced the electrochemical performance of SPAN cathode. Furthermore, the interwoven long‐rang fibers structure could accelerate the charge transfer and accommodation the volume expansion in the redox processes. Except for SPAN, to the best of our knowledge, there are no other types of organosulfur polymer cathode materials coupled with electrospinning that have been used in the Li–S system, but we believe organosulfur polymer with fibers structure could greatly enhance the electrochemical performance in Li–S batteries and it will happen soon.

### 5.2 Interlayer

Recently, many researchers have been paying attention to the functional separators or interlayers between separator and electrode, which is regarded as a potential strategy for restricting the LiPSs shuttle and promoting LiPS participation in the electrochemical reactions inside the sulfur cathode.^[^ ^84^ ^]^ Coincidentally, the lightweight and self‐supporting nature of the electrospinning‐based nanofibers membrane made it an appropriate choice of interlayer materials. In the Li–S battery system, the interlayers reported by researchers could be divided into two main categories: an interlayer between the separator and cathode working for the suppression of "shuttle effect" and the catalytic effect for the LiPS conversion; the interlayer between the separator and lithium anode functioning as a Li^+^ ions transmitter that inhibits the growth of lithium dendrites. In this part, we would focus on the interlayer used on the cathode side, while the interlayer for the lithium anode would be discussed in the follow‐up part concerning the anode.

**Figure 15:**
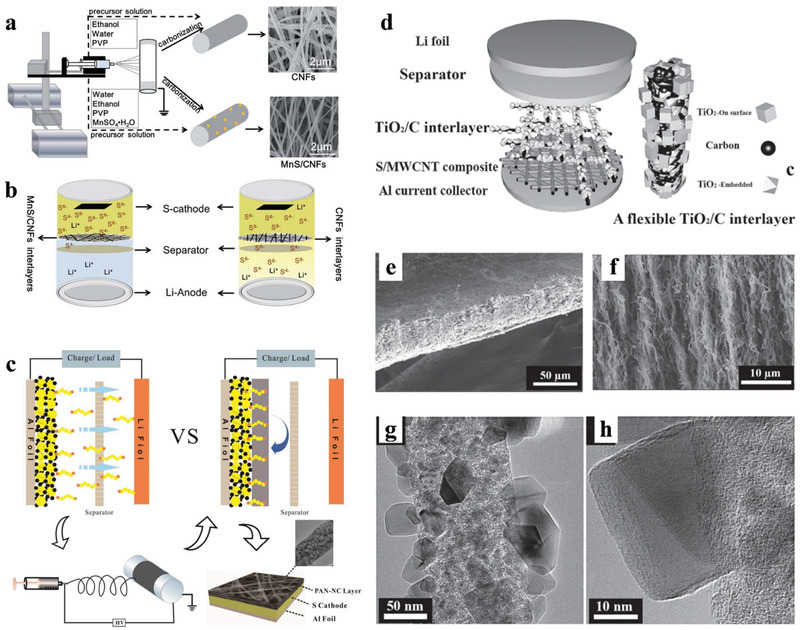
a) Schematic of the synthesis of the MnS/CNF and CNF interlayer. b) Schematic of different interlayers suppressing the diffusion of polysulfides. Reproduced with permission.^[^ ^86^ ^]^ Copyright 2020 the Royal Society of Chemistry. c) Schematic illustration for the cell configurations and fabricated process of PAN‐NC@Cathode. Reproduced with permission.^[^ ^87^ ^]^ Copyright 2017, American Chemical Society. d) Schematic of the Li–S battery configuration with a flexible interlayer. e,f) Cross‐sectional SEM images of the interlayer. g,h) TEM images of the interlayer. Reproduced with permission.^[^ ^88^ ^]^ Copyright 2017, Wiley‐VCH.

The 2D materials with layered structure as graphene and MXene were widely used in the interlayer of Li–S batteries for their outstanding LiPS interception ability.^[^ ^85^ ^]^ For the fabrication of such graphene or MXene, the 2D materials need to be dispersed and coated onto the surface of the sulfur cathodes or separator, making the structure and functions less controllable. For comparison, the free‐standing electrospinning‐based nanofiber membrane with 3D network structure not only showed excellent electronic conduction and controlled porous architecture to prevent the diffusion of LiPS, but also exhibited conclusive feasibility in combining functional groups for extra chemical adsorption and catalyst abilities, demonstrating that the electrospinning membrane could be a more suitable choice for the interlayers of Li–S cells. Wang et al.^[^ ^86^ ^]^ reported MnS/CNFs nanofibers synthesized by an electrospinning technique as a flexible interlayer for Li–S batteries, as shown in **Figure** **15**a, b. The introduced MnS particles were uniformly embedded in the carbon nanofibers, and at the same time, they brought nanopores into the carbon nanofibers substrate, which allowed the MnS particles to better contact with LiPSs for chemical adsorption. Associating the catalysis and adsorption effect to LiPSs of the MnS particles with the physical interception function of the free‐standing carbon nanofiber membrane, the cells with MnS/CNF interlayers achieved outstanding capability in a wide temperature range and low self‐discharge. In addition, Peng et al. developed a method to coating the PAN and nitrogen‐doped carbon black fiber (PANNC) directly onto the pure sulfur cathode through electrospinning technology.^[^ ^87^ ^]^ As is shown in Figure 15c, the conductive NC particles embedded in flexible PAN nanofibers tightly adhered to the surface of the sulfur cathode, which is not only proper for the electrolyte infiltration and Li^+^ diffusion but capable to buffer the volume expansion of the sulfur cathode. Hence, this PAN‐NC@Cathode reveals enhanced active materials utilization, rate capability, and cycling stability, indicating the promising application of this novel electrospinning‐based interlayer strategy. Vasant Kumar's group^[^ ^88^ ^]^ reported a praline‐like flexible interlayer consisting of TiO_2_ nanoparticles and CNFs to suppress the shuttle effect in Li–S batteries, achieved cycle stability and rate capability, as shown in Figure 15d–h. Carbon nanofibers materials could improve the conductivity of sulfur and Li_2_S, but the weak interaction between LiPS and nonpolar carbon cannot sufficiently suppress the shuttle effect. Therefore, the author coupled polar TiO_2_ nanoparticles into a carbon nanofiber framework to improve the adsorption ability of LiPS. This is a representative work using electrospinning to regulate the structures and components of nanofibers, greatly promoting the improvement of Li–S batteries.

### 5.3 Separator and Solid‐State Electrolytes

#### 5.3.1 Separator

As an essential part of the battery systems, the separator acts as an isolation zone between anode and cathode but provides a special channel for the electrolyte and ions. In the battery system containing Li‐ions, the separator is usually a porous polymer membrane that has both electrical insulation nature and transport channels for Li‐ions. Taking the notorious "shuttle effect" into consideration, the separator served in the Li–S battery system has been required more rigorous demands to suppress the LiPSs shuttle. More importantly, the safety of the battery system is highly relevant to the quality of the separator, hence the stability and heat resistance of the separator are also worth researching.^[^ ^84^, ^89^ ^]^ To sum up, an ideal separator for Li–S cells are demanded to have the following merits: 1) excellent insulation nature to avoid the internal short‐circuit; 2) high wettability and good permeability to ensure adequate electrolyte penetration and fast Li^+^ transport; 3) reliable functions in intercepting the LiPSs and confining them in the cathode side; 4) high electrochemical and thermal stability for long‐term safety. The current commercial Celgard polypropylene (PP) separator used in the Li–S batteries shows the advantages of uniform pores distribution, great chemical stability, and good mechanical strength. However, the PP separator is incapable of preventing the "shuttle effect" as well as resisting a harsh high temperature. Taking the physical interception effects of the cathode and interlayer designs into consideration, modification of the separators has been considered as an excellent alternative strategy to inhibit the LiPSs shuttle. The researchers have developed multiple strategies to improve the LiPS interception performance of the PP separator, concluding the adoption of various metal compounds,^[^ ^90^ ^]^ MOF,^[^ ^91^ ^]^ carbon‐based coatings,^[^ ^17a^ ^]^, and polymers containing polar groups^[^ ^92^ ^]^ directly coating on the PP separator as a syncretic layer. Yu et al.^[^ ^93^ ^]^ proposed a novel method to tailor the pore size of the traditional PP separator through a PIN. The PIN was successfully embedded into the micrometer‐scale pores of the PP separator after submerging the PP into the PIN solution. The PIN could suppress the penetration of LiPSs but allow the transport of Li^+^ ions, therefore the Li–S batteries with the PIN modified PP separator reveal improved electrochemical performance. The modification of the PP separator could easily enhance the Li–S battery electrochemical performance to a certain degree, but the inherent defects of PP separators as the poor thermal stability cannot be avoided, which remains to be a big problem, intensively hindering the practical applications of Li–S batteries.

**Figure 16:**
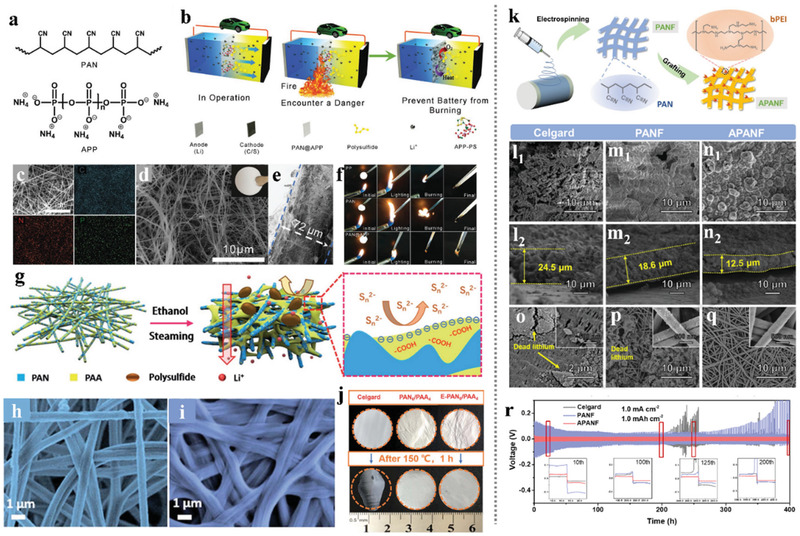
a) Chemical structures of PAN (top) and APP (down). b) Schematics of the multifunctional electrospinning PAN@APP separator with thermal‐triggered flame‐retardant properties for Li–S batteries. c) Elemental mapping images of C, N, and P in the selected region of PAN@APP. d,e) SEM images and cross‐section images of the PAN@APP membrane. The inset picture shows the digital photo of the PAN@APP separator. f) Flame‐retardant properties of PP, PAN, and PAN@APP separators. Reproduced with permission.^[^ ^57^ ^]^ Copyright 2018, Wiley‐VCH. g) Schematic diagram of the design flow for E‐PAN/PAA and the blocking mechanism against polysulfide. h,i) SEM images of PAN_6_/PAA_4_ and E‐PAN_6_/PAA_4_. j) Thermal shrinkage after exposure at 150 °C for 1 h of different membranes. Reproduced with permission.^[^ ^94^ ^]^ Copyright 2019 the Royal Society of Chemistry. k) A schematic of preparation of APANF. Top‐view and cross‐sectional SEM images of Li plating on Cu foil with l) Celgard, m) PANF, and n) APANF separators after 50 cycles. The morphology of o) Celgard, p) PANF, q) APANF, after cycling. The insets are high‐resolution images. r) Voltage profiles of symmetrical cells with different separators. Reproduced with permission.^[^ ^95^ ^]^ Copyright 2020, Elsevier.

The electrospinning‐based nanofiber membranes possess inherent porosity characteristics and show splendid flexibility in the composition adjustment or modification. Therefore, some novel electrospinning membranes have been developed as separators for improved thermal stability and efficient polysulfide inhibition. Lei et al.^[^ ^57^ ^]^ introduced a low‐cost flame retardant ammonium polyphosphate (APP) into the PAN precursor solution and fabricated a smart microfiber separator (PAN@APP) through electrospinning technology. As shown in **Figure** **16**a–f, the abundant amine groups and the phosphate radical in the APP showed strong binding interaction with LiPSs, thus realizing a charge repulsion effect and suppressing the transport of the negatively charged LiPSs to the anode side. Besides, the combination of PAN and APP could also avoid the direct contact between the APP and electrolyte, which could damage the electrochemical performance of the battery. The facile electrospinning technology realized a close combination of the two distinctive polymers of PAN and APP, thus constructing a multifunctional PAN@APP separator with the common merits of the two polymers. Compared with the traditional PP separator and electrospinning PAN separator, this multifunctional PAN@APP separator showed an improved performance in the Li–S battery, while overcoming the well‐known negative effects of fire‐retardant additives.

The micropores in the electrospinning‐based nanofibers films were generally formed from the disorder interweaving of the nanofibers, which made it troublesome in controlling the porosities to an appreciated level. In this respect, Zhu et al.^[^ ^94^ ^]^ developed a novel strategy to control the pore structure of electrospinning‐based nanofibers membrane. Through a facile electrospinning method, a polyacrylonitrile/poly (acrylic acid) (PAN/PAA) composite nanofibers membrane was prepared. As shown in Figure 16g–j, after an in situ ethanol vapor treatment, the electronegative PAA could be in situ extracted out from the electrospinning PAN/PAA nanofibers substrate, thus regulating the pore size into an appropriate level. The ethanol vapor‐treated PAN/PAA (E‐PAN/PAA) separator exhibited both physical interception and chemical barrier function to the LiPS, which were respectively benefited from the unique pore structure and the abundant electronegative carboxyl groups (—COOH). Therefore, the optimized E‐PAN_6_/PAA_4_ separator can not only efficiently prevent the "shuttle effect," but also endow the fast transport of Li^+^, resulting in a high discharge capacity of 1231 mAh g^−1^ at 0.1C, a better rate capacity of 487 mAh g^−1^ at 3C, and excellent cycle stability at 1C with only 0.03% of capacity decay per cycle.

The separator design should not only pay attention to the typical "shuttle effect" of the LiPS stem from the sulfur cathode but the complicated problems regarding the unstable metallic lithium anode also need to be considered. The inhomogeneous deposition of the Li^+^ ions and the unstable SEI layer would cause the notorious dendrite formation, which is lethal to the safety of the battery. In this respect, Hu et al.^[^ ^95^ ^]^ developed a functional ammoniated PAN nanofiber separator (APANF) to realize the inhibition of Li dendrite formation as well as the blocking of LiPS shuttling. As shown in Figure 16k–r, the multifunctional APANF separator was fabricated by a facile electrospinning method coupled with the following chemical grafting of the polyethyleneimine (PEI). The cross‐linked nanofiber structure and the polar surface groups acted as a Li^+^ ions regulator to promote the Li^+^ ions depositing in a more homogeneous 3D spherical deposition pattern, and the free ammonia groups in favor of the Li_3_N‐rich SEI layer generation, further suppressing the Li dendrite growth. Besides, the APANF also showed a robust chemical adsorption ability with LiPSs, thus effectively restricting the shuttling effect. Therefore, the Li/Cu half cells with APANF separator achieved a high stable Coulombic efficiency of 98% for 120 cycles, and the Li–S cell with APANF separator also showed much outstanding cycling stability and long‐life span than those with Celgard PP separator.

**Figure 17:**
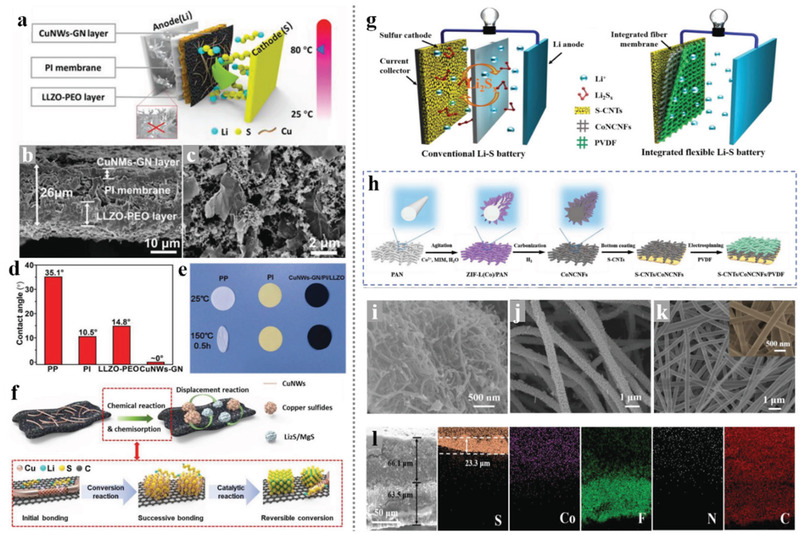
a) Schematic illustration of the cell configuration based on the CuNWs‐GN/PI/LLZO separator. b) Cross‐section SEM image of the CuNWsGN/PI/LLZO separator. c) SEM image of the CuNWs‐GN surface on polyimide membrane. d) The electrolyte contacts angle of varied separators. e) The thermal stability of varied separators at 150 °C for 0.5 h. f) Schematic illustration of the chemical anchoring and catalytic functionality of the copper nanowires‐graphene coating layers toward soluble polysulfide species (top picture: macroscale changes at the electrode level and down: microscale change at the molecular level). Reproduced with permission.^[^ ^96^ ^]^ Copyright 2020, Wiley‐VCH. Schematic illustrations of g) the working principle of the conventional Li–S battery system and integrated flexible Li–S battery system, h) the fabrication of the integrated three‐in‐one fibrous membrane. SEM image of i) S‐CNTs, j) CoNCNFs layer, and k) PVDF fibrous layer. l) Cross‐sectional SEM image and EDS mappings of the three‐in‐one S‐CNTs/CoNCNFs/PVDF membrane. Reproduced with permission.^[^ ^35^ ^]^ Copyright 2019, Wiley‐VCH.

For the further improvement of a multifunctional separator, Zhou's researcher team proposed a novel strategy for integrating several distinctive materials into an independent separator with a sandwich architecture and integrated functions.^[^ ^96^ ^]^ As shown in **Figure** **17**a–f, the integrated separator is composed of the electrospinning polyimide (PI) nonwovens, the CuNWs‐GN coating film, and an external coating of LLZO‐PEO film. In this novel hierarchical separator, the PI nonwovens with intrinsically 3D structure stability, unique flame‐retardant property, and good mechanical strength served as a basic skeleton; the CuNWs‐GN layer with 3D stacked structure and the subtle reaction conversion with LiPSs endowed efficient shuttle effect blocking ability including both physical interception and strong chemical bonding effects; the rigid LLZO‐PEO solid‐state electrolyte layer can effectively suppress the dendritic metal growth and improve the safety of batteries. Therefore, at an elevated temperature of 80°C, pure cathodes with high sulfur loading and a relatively low E/S ratio delivered a high initial discharge capacity and superior cycling stability, as well as a progressive performance in the Mg–S battery system. The electrospinning polymer nanofibers membranes were regarded as desirable candidates of the separator material in Li–S batteries for their natural 3D network structure and outstanding flexibility, while the carbonized electrospinning polymer nanofibers membranes were ideal sulfur host materials for their remarkable conductivity and porosity. Ding's research team proposed a novel strategy that integrating the electrospinning polymer nanofibers membrane and electrospinning carbon nanofibers membrane with a typical sulfur cathode into a multifunctional three‐in‐one fibrous membrane.^[^ ^35^ ^]^ As shown in Figure 17g–l, the three‐in‐one fibrous membrane was fabricated by a facile and effective integration strategy based on electrospinning and base‐coating techniques. The inner coating layer as the sulfur source was a close‐packed mixture in which sulfur was spatially embedded in numerous conductive CNTs; the middle layer was constituted by the Co and N codoped PAN‐derived hierarchical carbon nanofibers (CoNCNFs) with binary hierarchical architecture which showed excellent surface/chemical affinity toward the LiPSs and could mitigate the LiPS shuttle; the outer layer (closest to the Li anode) serving as a high‐flux polymer separator was a typical electrospinning polyvinylidene fluoride (PVDF) nanofibrous film. Due to the distinguished and distinctive abilities of each layer and their synergistic effects of the integrated flexible S‐CNTs/CoNCNFs/PVDF membranes, the cell under high sulfur loading of 13.2 mg cm^−2^ with an E/S ratio of 6 mL g^−1^ still exhibited high areal capacity of 11.4 mAh cm^−2^ and exceptional folded application performance.

In addition to considering the fundamental functions as the basic component in the batteries, the separator materials also show great prospects in developing additional functions targeting different application scenarios, in which researchers have made many efforts and harvested a lot. In Li–S batteries, the electrospinning‐based nanofibers membrane, the metal‐organic framework (MOF) based membrane,^[^ ^97^ ^]^ and other novel membrane has been developed to replace the current commercial PP separator, which is incapable of inhibiting LiPSs shuttle and coping with the lithium metal anode. The electrospinning‐based nanofibers membrane possesses the natural 3D network and porous structure and has been regarded as a promising candidate for separator materials. Moreover, with its extremely strong controllability and feasibility, the electrospinning materials show great potential in combating the cruel problems in the Li–S batteries.

#### 5.3.2 Solid‐State Electrolytes

An electrospinning separator or modified electrospinning separator is an effective strategy to restrain the shuttle phenomenon in Li–S batteries. However, in a liquid electrolyte system, the shuttle effect cannot be fully suppressed, due to the high solubility of long‐chain sulfur species and concentration polarization in Li–S systems. In addition, the electrochemical and thermal instabilities in the liquid electrolyte‐based Li–S system result in more potential safety issues which are from the flammability of organic electrolytes and the overgrowth of lithium dendrites. Recently, adopting all‐solid‐state electrolytes to replace liquid electrolytes to suppress the shuttle effect and improve the electrochemical and thermal instabilities in all‐solid‐state Li–S batteries becoming a promising strategy. Therefore, many kinds of solid‐state electrolytes have been exploited in all‐solid‐state Li–S batteries, such as gel polymer electrolytes, ceramic electrolytes, and hybrid electrolytes, deeply promoting the advance of the Li–S system. More importantly, the use of solid‐state electrolytes replacing the liquid electrolytes and separators could decrease the total weight and increase the energy density of batteries.

**Figure 18:**
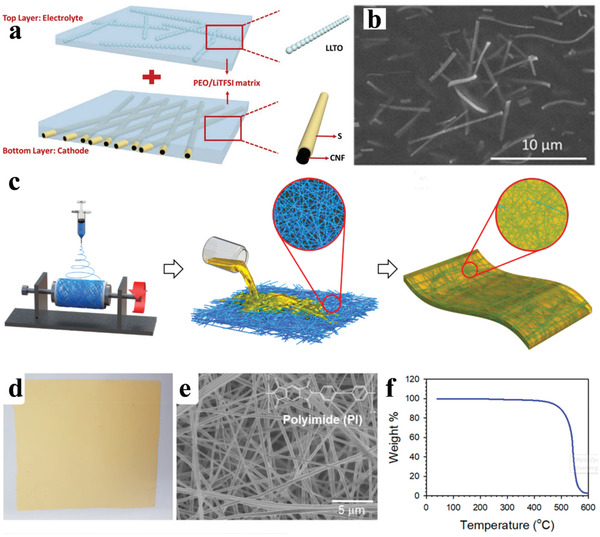
a) Schematic illustration for the preparation of PEO/LLTO solid electrolytes and S/CNF cathode. b) SEM image of PEO/LLTO solid electrolytes. Reproduced with permission.^[^ ^98^ ^]^ Copyright 2019, Elsevier. c) Schematic of the flexible solid electrolyte membrane. d,e) Thermal properties and flammability of the solid FRPC electrolyte. f) Schematic illustrating for the solid electrolytes by infiltrating PI nanofibers with solution Li_6_PS_5_Cl_0.5_Br_0.5_. Reproduced with permission.^[^ ^100^ ^]^ Copyright 20, American Chemical Society.

Among all kinds of solid electrolytes, electrospinning all‐solid‐state electrolytes into 1D nanofibers structure attract more attention due to the high ion transportability and the high porosity. Furthermore, the 1 D structured solid‐state electrolytes could embed in other polymer matrices to create a continuous ionic transport pathway and reduce the interfacial resistance. For example, Zhu et al.^[^ ^98^ ^]^ reported an electrospinning Li_0.33_La_0.557_TiO_3_ (LLTO) nanofiber embedded in poly (ethylene oxide) (PEO) solid composite serving as all solid electrolytes in all‐solid‐state Li–S batteries to improve the thermal stability and reduce the interfacial resistance, as shown in **Figure** **18**a,b. In this work, a completely suppressed shuttle effect, enhanced ionic transport pathways, reduced interfacial resistance, and an improved amorphous region in the PEO matrix were obtained, greatly promoted the electrochemical performance of the all‐solid‐state Li–S batteries. In 2016, Hu et al.^[^ ^99^ ^]^ first reported a 3D Li^+^ conducting network based on electrospinning Li_6.4_La_3_Zr_2_Al_0.2_O_12_ (LLZO) ceramic nanofibers embedded in PEO matrix serving as solid‐state electrolytes in lithium metal batteries. These all‐solid membranes exhibited a high ionic conductivity and stable cycle life in Li symmetric batteries, due to the effective blocking of the overgrowth of lithium dendrites. Furthermore, in typical all‐solid‐state batteries for practical applications, a thickness of <100 µm for solid electrolytes is needed to achieve high energy density batteries. Therefore, Lee et al.^[^ ^100^ ^]^ reported a thin thickness of 40–70 µm and flexible solid electrolyte for all‐solid‐state Li‐ion batteries, which achieved excellent thermal stability up to 400 °C and outstanding electrochemical performance of 146 mAh g^−1^, as shown in Figure 18c–f. In this study, electrospinning polyimide (PI) nanofibers were first prepared to offer a unique porous structure and ultrahigh thermal stability to accommodate the highly conductive infiltrating solution Li_6_PS_5_Cl_0.5_Br_0.5_. This work demonstrated a new proof‐of‐concept of fabrication protocol for all‐solid‐state Li‐ion batteries. In conclusion, these works prove that the method of electrospun solid‐state electrolytes into nanofibers structure could enhance thermal stability and increase the energy density of batteries.

### 5.4 Anode

The graphite anode used in current lithium‐ion batteries has almost reached its theoretical limit, which critically hinders the further extension of the battery energy density.^[^ ^101^ ^]^ Therefore, various materials have been deeply studied as anode materials for lithium‐ion batteries, such as carbons, metals/semiconductors, metallic compound, and their combinations, due to their potentials in delivering extraordinarily higher capacity compared with a graphite anode. Besides, many researchers also expressed an ever‐growing interest in revisiting lithium metal anode, as the body‐centered cubic (BCC) lithium metal has the lowest electrochemical potential (‐3.04 V vs NHE) and the unparalleled highest capacity (3860 mAh g^−1^) among the existing known anode materials.^[^ ^102^ ^]^ With the Li metal as an anode, the Li–S batteries also need to face several issues accompany with the Li metal, such as the overgrowth of Li dendrite, large volume fluctuation, and the structural and chemical irreversibility in the electrolyte. These problems not only encumbered the cycle stability but also bring serious security risks into the batteries. During the charge and discharge process of the batteries, the intercalation and deintercalation of the Li^+^ from the surface of the Li metal were disordered, resulting in the disordered growth of Li metal crystals. Meanwhile, the limited surface area of the copper foil current collector could not afford enough Li metal and navigate them into stable deposition. On the other hand, due to the host‐less nature of the plating of Li, the SEI required for restricting side reactions would be repeatedly destroyed, leading to a vicious cycle of anode degradation.

**Figure 19:**
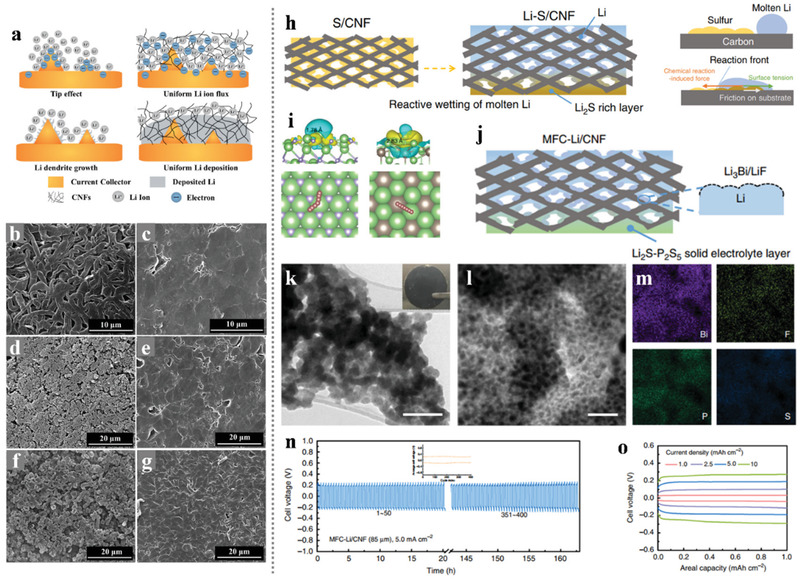
a) Schematic illustrations of the Li dendrite growth on a bare current collector and the uniform Li deposition on a current collector with a CNF interlayer. SEM images of the Li deposition on a bare Cu foil at current densities of b) 0.5 mA cm^−2^, c) 1 mA cm^−2^ and d) 2 mA cm^−2^, respectively. SEM images of the Li deposition on a Cu foil with a CNF interlayer at current densities of e) 0.5 mA cm^−2^, f) 1 mA cm^−2^ and g) 2 mA cm^−2^, respectively. Reproduced with permission.^[^ ^107^ ^]^ Copyright 2017, Elsevier. h) A cartoon illustrating the fabrication process of Li–S/CNF. i) Charge density difference plots showing the adsorption of a Li adatom on the substrate of LiF (111) (left) and Li_3_Bi (111) (right) (top row); the diffusion pathways of Li adatoms on the corresponding surfaces (bottom row). The green, purple, and brown balls represent lithium, fluorine, and bismuth atoms, respectively. j) A cartoon showing the coating strategy to derive MFC‐Li/CNF. k) TEM image of the protective layer stripped from the MFC‐Li electrode. l,m) TEM/EDS analysis of the protective layer. n) Representative cycling voltage profiles at 1.0 mAh cm^−2^ at 5.0 mA cm^−2^ for the symmetric cell assembled with the MFC‐Li/CNF electrode, the inset shows the average cell voltage versus the cycle index. o) Voltage profiles at different current densities. Reproduced with permission.^[^ ^108^ ^]^ Copyright 2019, Nature.

To address the above‐mentioned problems, researchers have taken great efforts and proposed many constructive strategies. For instance, Ding et al.^[^ ^103^ ^]^ and Guo et al.^[^ ^59^ ^]^ attempted to optimize the electrolyte compositions and additives to homogenize the Li^+^ flux to induce uniform Li deposition and enhance the stability and uniformity of the SEI on the anode surface. Besides, solid electrolytes and various mechanical barriers^[^ ^104^ ^]^ with high shear modulus were also explored for suppressing the growth of Li dendrite. In recent years, the construction of a 3D framework with lithium affinitive materials has been regarded as a feasible and efficient route to improve the stability of the Li metal anode. The 3D interconnected architectures not only act as a natural transportation network for both electrons and Li^+^ ions, but also provide a large specific surface area, reducing the current density and homogenizing the distribution of the positive charges, and thus ensuring uniform and stable deposition of lithium metal. For example, Cui et al.^[^ ^105^ ^]^ reported a flexible, interconnected, hollow amorphous carbon nanosphere coating as a stable SEI which could prevent the penetration of solvent molecules in the electrolyte but allow the transfer of Li^+^ ions. Besides, the Li^+^ ions passing through the SEI were selectively deposited on copper foil due to the conductivity differences between carbon nanospheres and copper collector, thus avoiding the damage to the surface SEI and the growth of Li dendrite. Among the various 3D porous carbon materials, the CNFs have been regarded as ideal host materials for their 3D cross‐linked conducting networks, which can facilitate the transportation of electrons and Li^+^ ions allow Li to deposit on the surface of the network. Zhang et al.^[^ ^106^ ^]^ reported an impressive work that using the vacuum‐filtrated CNFs film to modify the Cu current collector and achieve dendrite‐free Li metal deposition in the anode. Sun's research team developed a novel strategy that coats the cellulose aerogel‐derived CNFs on both sides of the separator to deal with the many problems in both of sulfur cathode and the Li anode.^[^ ^107^ ^]^ As shown in **Figure** **19**a–g, the conductivity CNFs network was able to redistribute the electrons and avoided the inhomogeneous distribution of Li^+^, thereby inhibiting the generate and overgrowth of Li dendrites on the surface of Cu foils. Moreover, the CNFs network could also render a uniform Li^+^ flux near the anode surface and lead to a smooth Li deposition with its porous architecture. As a result, the CNF‐modified Li metal anodes delivered enhanced Coulombic efficiencies, as well as the Li–S battery with CNF interlayers, showed superior capacity and cycle life.

Compared with other methods for CNFs manufacturing, the electrospinning technology shows plenty of advantages such as low cost, high efficiency, scalability, and natural film‐forming properties, thereby the electrospinning CNFs exhibit great potential in constructing a modification layer for Lithium depositing. Moreover, the Li metal has a low melting point of 180 °C which can be infused into a 3D matrix and construct a composite Li anode. The free‐standing electrospinning CNFs films happen to be well qualified for the liquefied Li metal infusion process, which is similar to the construction of a free‐standing sulfur cathode using the melt diffusion method. However, the direct thermal infiltration of molten Li always encountered the surface lithiophobicity issue, blocking the Li infusion into the CNFs matrix. To tackle this issue, Ren et al.^[^ ^108^ ^]^ developed a novel reactive wetting strategy to actuate the Li infusion with a reaction between Li and sulfur. As shown in Figure 19h, the sulfur was selected as a surfactant to pretreat the CNFs to overcome friction and surface tension towards the molten Li metal. Moreover, a feasible strategy was also proposed to construct a robust SEI film for this porous Li/S‐CNF scaffold. After a spontaneous reaction between Li with a mixture of BiF_3_–P_2_S_5_ complex, artificial SEI films were generated onto the Li‐coated nanofibers. Subsequently, an annealing treatment to the polymerized Li_2_S–P_2_S_5_ composite could form a uniform solid electrolyte layer and cover the surface of the Li scaffold. As illustrated in Figure 19i–m, after the above two‐step treatments, a metal fluoride complex protected Li/CNF (MFC‐Li/CNF) anode with porous structure and the double functional protective layer was fabricated. Figure 19n, o exhibits that the symmetric cells with the MFC‐Li/CNF electrode could sustain more than 400 cycles even at a current density of 5.0 mA cm^−2^ and revealed smaller overpotentials from 1.0 to 10 mA cm^−2^. Moreover, the Li–S full battery with this MFC‐Li/CNF anode also delivered stable cycling performance with a high areal capacity above 7.5 mAh cm^−2^ even at a current density of 4.0 mA cm^−2^.

**Figure 20:**
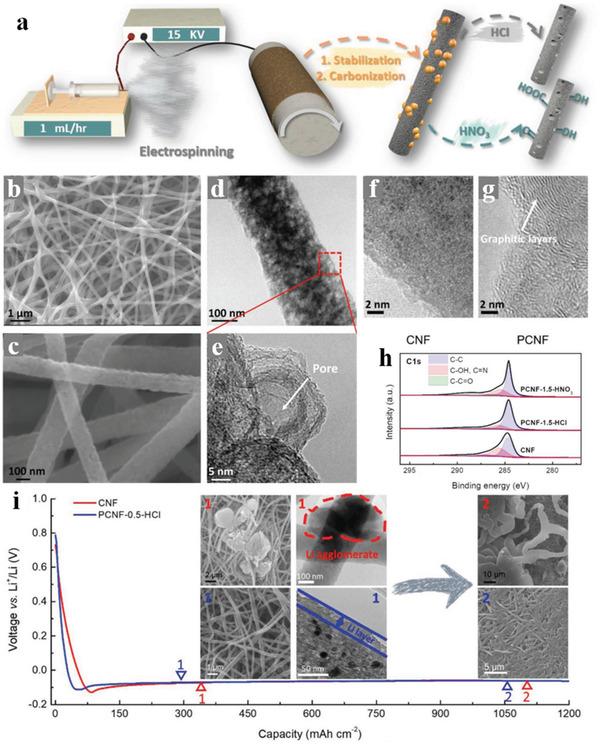
a) Schematic representation of the synthesis of PCNFs with or without oxygenated functional groups. b) Low and c) high‐magnification SEM images of PCNF‐1.5‐HNO_3._ d) TEM image and e) HRTEM image of PCNF‐1.5‐HNO_3_. HRTEM images of f) neat CNF and g) PCNF‐1.5‐HNO_3_ showing distinct degrees of graphitization. h) Deconvoluted C1s spectra of PCNFs prepared with different etching agents and neat CNFs. i) Ex situ SEM and TEM images of the neat CNF and PCNF electrodes at different stages of Li plating. Reproduced with permission.^[^ ^109^ ^]^ Copyright 2018, Wiley‐VCH.

**Figure 21:**
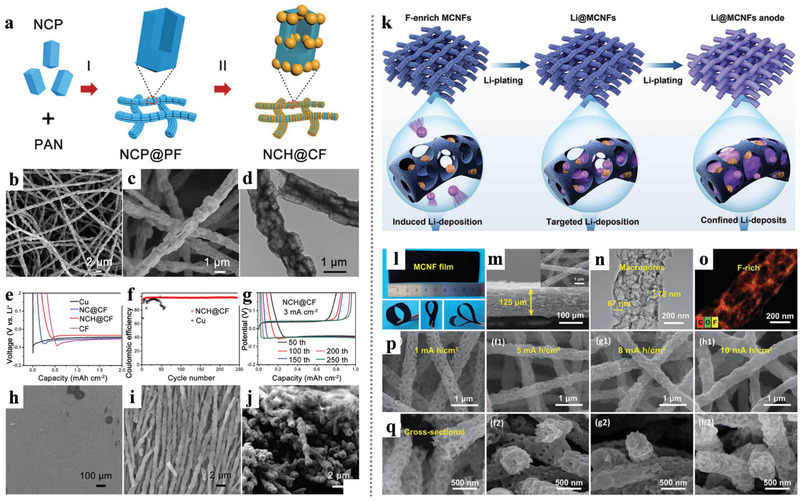
a) Schematic illustration of the fabrication procedure of NCH@CFs. b,c) FESEM and d) TEM images of NCH@CFs. e) Voltage profiles of the Li plating on different hosts. f) CE and the g) voltage profiles of the NCH@CF and Cu hosts. h–j) FESEM images of the NCH@CF host with a plating capacity of 6 mAh cm^−2^. Reproduced with permission.^[^ ^40^ ^]^ Copyright 2021, Wiley‐VCH. k) Illustration of lithium deposition on the MCNFs. l) Optical images of the MCNFs. m) Cross‐section and top‐view SEM images of MCNFs. n) TEM image and o) EDS mapping of MCNFs. p,q) Top‐view and cross‐section SEM images of the MCNFs with plated lithium of 1–10 mAh cm^−2^. Reproduced with permission.^[^ ^110^ ^]^ Copyright 2021, Royal Society of Chemistry.

Although the surface treatment could drive reactive wetting of molten Li on the CNFs surface and improve the lithium affinity toward the CNFs scaffold, the Li plating process on the carbonaceous materials was also worth exploring when preparing reliable Li/CNFs anode. However, the correlation between Li plating behavior and the surface characteristics of carbonaceous materials rarely attracts the researcher's attention. In this case, Kim's research team proposed an enlightening work that elaborated the mechanism regarding Li plating behaviors on the porous electrospinning carbon nanofibers.^[^ ^109^ ^]^ As shown in **Figure** **20**, the porous carbon nanofibers (PCNFs) with a high graphitization degree were fabricated through a novel templet method ingeniously using the catalytic Fe_3_C particles as the templet. The HNO_3_ and HCl solution with diverse concentrations were selected as etchant and source of the oxygenated functional groups. The experiments and theoretical calculations together demonstrated that the open pores created in the CNF surface could prompt the controlled initial nucleation of Li, as well as the introduction of oxygenated functional groups could significantly ameliorate the uniform nucleation and growth of Li on the PCNFs surface due to the largely reduced energy barrier required for the growth of Li nuclei. Moreover, the optimized Li‐plated PCNF‐0.5‐HNO_3_ anodes were assembled into Li–S batteries with a sulfur‐infiltrated carbon cathode, the full cells delivered much elevated electrochemical performance compared with the one that uses the Li‐plated stainless steel as an anode. Furthermore, many other electrospinning PCNFs with plenty of lithiophilic sites were developed to induce homogenous Li deposition. Fang et al.^[^ ^42^ ^]^ developed a facile method by electrospinning to build Ag‐doped carbon microporous fibers (Ag@CMFs) as a 3D lithium host for stable lithium metal batteries. The Ag‐doped nanoparticles greatly enhanced the lithium affinity of CMFs, which could effectively regulate the Li deposition progress in lithium metal batteries. Furthermore, the 3D microporous fiber structure could decrease the current density and accommodate the volume fluctuation during the Li^+^ plating/stripping process in the lithium anode. As a result, a high CE of 98% was achieved for 800 cycles, even at a high current density of 5 mA cm^−2^ can still be achieved in 200 cycles with a smooth and dense surface of lithium anode. Chen et al.^[^ ^40^ ^]^ reported a novel self‐engaged method to make Ni‐Co hollow prisms@carbon fibers as 3D lithium host to induce homogenous Li deposition, the materials preparation procedure is illustrated in **Figure** **21**a. In the composite, the Ni‐Co is evenly distributed in the CNFs serving as the lithiophilic nucleation sites to induce the homogenous Li deposition and the N species in the CNFs could enhance the lithium affinity of the carbon skeleton. In addition, plenty of voids with high curvature could regulate the electric field and the hollow prisms greatly increase the volume utilization in the Li plating/stripping progress. Therefore, the 3D NCH@CFs could serve as an efficient lithium host and delivered a low nucleation overpotential, high CE, and stable cycle life. Zhao et al.^[^ ^110^ ^]^ reported a special fluoride‐rich CNFs serve as lithiophilicity lithium, enabling an outstanding electrochemical performance host in lithium–metal batteries, as shown in Figure 21k–q. More importantly, this work explored the influence of the pore size from the CNFs frameworks on the deposition of lithium, enriched the mechanism of lithium deposition on CNFs.

**Figure 22:**
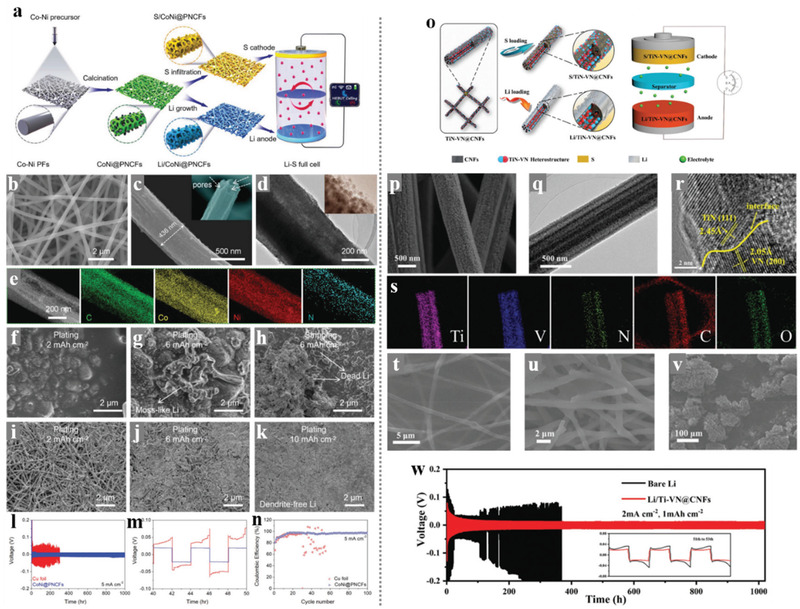
a) Schematic diagram of synthetic procedures of CoNi@PNCFs electrode and an electronic watch powered by Li–S full cell. b,c) SEM images of CoNi@PNCFs. d,e) TEM images and elemental mapping images of CoNi@PNCFs. f–h) SEM images of Cu electrode after 50 cycles of Li plating/stripping, i–k) CoNi@PNCFs electrode after 50 cycles of Li plating/stripping. l) Long‐term cycling performance of Cu and CoNi@PNCFs electrodes at current densities of 5 mA cm^−2^ and capacity of 10 mAh cm^−2^, m) between 40 and 50 h, and n) CE of CoNi@PNCFs and Cu electrodes. Reproduced with permission.^[^ ^36^ ^]^ Copyright 2020, Wiley‐VCH. o) Synthesis schematic for the S/TiN‐VN@CNFs cathode, the Li/TiN‐VN@CNFs anode, and the S/TiN‐VN@CNFs || Li/TiN‐VN@CNFs full battery configuration. p) SEM images, q) TEM image, r) High‐resolution TEM (HRTEM) images, and s) TEM elemental mapping images of the TiN‐VN@CNFs. t) SEM images of the Li/TiN‐VN@CNFs. SEM images of u) the Li/TiN‐VN@CNFs and v) the bare Li electrodes after 100 cycles at 1 mA cm^−2^ with a fixed capacity of 1 mAh cm^−2^. w) Cycling performance between bare Li and Li/TiN‐VN@CNFs symmetrical cells. Reproduced with permission.^[^ ^2b^ ^]^ Copyright 2020, Wiley‐VCH.

The electrospinning PCNFs with a highly conductive network has shown great applicability in serving as hosts for both sulfur cathode and Li metal anode, this means that the elaborated PCNFs embedded with active catalysis and adsorption sites for mitigating the LiPS shuttling could also be employed in the Li metal anode to induce homogenous Li growth. Many electrospinning PCNFs embedded with diverse metal compounds have been proved to have marvelous flexibility and excellent mechanical strength, thus the full batteries using flexible PCNFs membranes as the hosts of both cathode and anode could realize dependable practicability in flexible devices. As shown in **Figure** **22**a–n, He et al.^[^ ^36^ ^]^ reported an all‐purpose CoNi@PNCFs with multiple adsorptive/catalytic sites that can simultaneously serve in the sulfur cathode and Li metal anode. The CoNi metals and metal–N–C sites within the skeleton of mesoporous carbon could not only offer improved interactions with LiPSs as absorption and catalysis effect but also induce homogenous Li nucleus growth when cooperating with the 3D network structure. A Li–S full cell was assembled with S/CoNi@PNCFs cathode and Li/CoNi@PNCFs anode, while the sulfur was infused in the CoNi@PNCFs membrane through a modified melt diffusion method, and the Li metal was plated into the CoNi@PNCFs scaffold. This full cell delivers a high specific capacity of 785 mAh g^−1^ and long cycle performance at 5 C with a sulfur loading of 1.5 mg cm^−2^ and Li plating around 6 mAh cm^−2^. Moreover, a highly flexible Li–S pouch cell using this all‐purpose CoNi@PNCFs as electrode hosts was demonstrated and evaluated, which is further illuminating in the application of flexible electronics. The transition metal nitride (e.g., TiN, VN, and MoN) and metal oxide (e.g., TiO_2_, V_2_O_5_, and MnO_2_) have been used as anchoring additives for their strong chemical adsorption for LiPS. Recently, researchers have found that the heterostructures born at the junction of the two different crystal lattices of metal compounds possess favorable electrocatalysis ability to accelerate LiPSs transformation. For instance, Zhou et al.^[^ ^111^ ^]^ reported a twinborn TiO_2_–TiN heterostructure loaded on the graphene as a separator coating layer to restrain the LiPS shuttling. Song et al.^[^ ^112^ ^]^ reported a similar investigation that the VO_2_–VN heterostructure as sulfur hosts could alleviate the LiPS shuttling and promote the redox kinetics in LiPSs transformation. Except for serving as sulfur hosts, the dual nitride heterostructure embedded electrospinning CNFs were also been proved to perform competently when serving as the Li metal hosts. As shown in Figure 22o–w, Yu and co‐workers proposed a dual‐functional flexible CNFs membrane in suit embedded with TiN‐VN heterostructure (TiN‐VN@CNFs) as supporting skeleton simultaneously for both of sulfur cathode and Li anode.^[^ ^2b^ ^]^ Since the TiN‐VN@CNFs possess a 3D CNFs network and evenly distributed TiN‐VN sites, the sulfur and Li metal could be infiltrated into the TiN‐VN@CNFs host via a molten diffusion method. The TiN‐VN@CNFs combined the merits of strong anchoring ability for LiPSs, catalysis effect towards the transformation of LiPS, the lithiophilic feature and the electron/ion flux regulation, thus considerably realize a superior rate performance, ultralong cycling life and nearly 100% Coulombic efficiency in a coupled Li–S full‐battery.

Porous carbonaceous materials have been demonstrated realizable as host materials for Li metal to address the obstacle regarding the uneven Li deposition and the accompanied Li dendrites growth. As a simple and novel technology, the electrospinning technique has been regarded as a precursor in fabricating free‐standing large‐scale porous carbonaceous films such as porous CNFs and their derivatives. Generally, the nondoped porous CNFs were not competent hosts of Li metal especially in a melt diffusion process for the less affinity between the fresh surface of carbon and the liquid Li metal. Hence, the surface treatment for the CNFs has been developed to prompt the Li‐infusing or Li‐plating process on the CNFs skeleton. Moreover, some electrospinning CNFs modified with the in‐site introduced metal compound nano sites have been demonstrated to be adequate Li metal hosts materials for their natural affinity to liquid Li metal or Li^+^ ions, which means the dual applicability of a specific electrospinning CNFs membrane in both sulfur cathode and Li metal anode of the Li–S batteries could be achieved. Besides, in addition to replacing the Cu foil current collector and directly serving as free‐standing hosts for the Li metal anode, the electrospinning CNFs network could also be used as a modified layer onto the current collector to guide the Li^+^ ions to form uniform lithium metal deposition, thus inhibit the growth of Li dendrite. To sum up, the electrospinning‐based nanofibers are promising in replacing the Cu current collector in Li metal anode, the novel Li/CNFs composite electrodes, and the Li anode modified with the nanofibers functional layer open a new window to address the inherent issues of Li anodes.

## 6 Conclusion and Perspective

The development of efficient and safe energy storage technology is the key part in solving the growing fossil energy crisis and the related environmental pollution. In recent years, numerous types of energy devices and novel energy storage materials have been widely used and evoked much interest from researchers around the world. Among them, the Li–S batteries have attracted much concern for their intrinsic ultrahigh energy density. Nevertheless, the poor conductivity of the active sulfur in the cathode, the "shuttle effect" of the intermediate LiPS, and the extremely unstable Li metal anode hindered the actual performance of Li–S batteries. In this case, researches concerning the development and modification of the materials about the cathode, interlayer, separator, and anode in Li–S batteries have been in full swing. And, miscellaneous novel materials have been developed and applied in each component of the Li–S battery system to function as hosts, functional additives, and modified layers. Electrospinning‐based nanofibers materials, including carbon nanofibers, polymer nanofibers, inorganic nanofibers, composite nanofibers, have been regarded as promising materials in many frontier fields for their large surface area, structural designability, component flexibility, adjustable defects or active sites, miraculous 3D structure, and flexibility. Unsurprisingly, the various electrospinning‐based nanofibers materials could be applied in the Li–S batteries. And research found that the functions of the electrospinning‐based nanofibers materials could be well‐matched with the key demands in Li–S batteries.

In this review, we have introduced the basic electrospinning technique and systematically illustrated its application potential in the Li–S batteries. When combined with other technologies such as CVD and hydrothermal methods, the diversity and functionality of electrospinning‐based nanofibers could be further expanded, thus enabling flexible and oriented application towards the various scenarios for battery materials. In the current research related to the Li–S batteries, the core components inside the battery could be mainly divided into the sulfur cathode, Li metal anode, separator, and interlayer. Besides, researchers also proposed some novel solutions such as lithium‐free anodes and using LiPSs as a sulfur resource. However, these methods all could not avoid the vital problems of the Li–S batteries because of the contradiction between battery reaction kinetics and the diffusion of LiPS, and the deposition placement of Li^+^ ions. By summarizing the recent advance in numerous electrospinning‐based nanofibers materials for Li–S batteries, we have separately elaborated on the application scenarios of the electrospinning‐based nanofibers materials in the main parts of the Li–S batteries as cathode, anode, separator, and interlayer.

On the cathode side, the conductive carbonaceous nanofibers have been regarded as the most appropriate choice for the poor conductivity of the active sulfur. Various structural adjustment approaches of the nano frameworks composed of nanofibers were developed and presented to ensure as much as possible sulfur encapsulated in the nano frameworks. Besides, polar groups were introduced into the nanofibers during the electrospinning process or accessional hydrothermal process to improve the adsorption and catalysis effect towards LiPSs, thus ameliorating the "shuttle effect." When serving as the interlayers, the self‐standing nature of electrospinning‐based nanofibers also displayed their advantages: 1) the porous 3D network structure acted as a natural physical interception layer; 2) the absorptive polar group embedded in the nano framework could effectively capture the LiPS from the electrolyte and retention them in nanopores as the reservoirs; 3) the independent design showed better freedom for eliminated the complicated composite process with the active sulfur. However, it was noteworthy that the additional‐introduced interlayers should be light and thin enough, otherwise, the actual energy density of the whole Li–S battery system would be affected. When applied in the separators of Li–S cells, the electrospinning polymer films also showed their superiority including lightweight, adjustable pore size, and thermal stability. However, the thickness of the electrospinning film is normally larger than that of commercial PP separators. Many types of research have been presented that the electrospinning‐based nanofibers perform well in restricting LiPSs within the cathode side, leading to better utilization of active sulfur and superior cycling life. We also reviewed the application strategies of electrospinning‐based nanofibers in the Li metal anode. The conductive 3D network structure of the electrospinning carbonaceous nanofibers could homogenize the charge distribution in the anode and guide the Li^+^ depositing into smooth Li metal, thus inhibiting the dendrite issues. And the surface treatment of electrospinning carbonaceous nanofibers has been proved to be efficient in improving the surface affinity to Li^+^ and liquid Li metal, which indicates that the Li metal could be confined into the electrospinning nano framework to construct a CNFs/Li anode through plating or melt diffusion method, which is similar with the sulfur hosts strategy in the cathode. When compared with the traditional Li anode coating on Cu foil current collector, these novel CNFs/Li anodes delivered a smoother Li deposition/stripping process as well as a better coulomb efficiency. Furthermore, some innovative material designs based on electrospinning‐based nanofibers such as hierarchical functional separator, integration of electrode and separator, and dual‐functional hosts for both cathode and anode have been developed in Li–S batteries.

The plenty of works reviewed in this paper have demonstrated the potential of electrospinning‐based nanofibers materials in promoting the practical application of Li–S batteries. However, it is worth mentioning that there are still some rigorous obstacles on the road of the commercialization of Li–S batteries, and the approaches of electrospinning‐based nanofibers electrodes still have their deficiencies waiting to be overcome. For instance: 1) The porous structure of nanofibers makes it difficult to achieve a high tap density of electrode, which is essential to improve the energy density of in the multilayer‐pouch cell; 2) In the view of industrial synthesis and commercial application, the poor production rate of melt diffusion and plating process results in a high cost of the nanofibers‐based electrode; 3) The reliable connection between porous nanofibers and alloys is still not easy in principle, this means the free‐standing nanofibers‐based electrodes need to cooperate with the additional Al/Cu foil, which could provide reliable tabs in the Li–S pouch cells, and this undoubtedly reduces the practical energy density of the battery.

Although there are still many challenges toward the practical application of Li–S batteries, the fast‐developing nanotechnologies like electrospinning have delivered optimistic waves that advanced material and structure could realize promising nanofibers‐based cathodes and anodes in Li–S batteries to enable next‐generation energy storage. This review is prepared to pave the way for the core fields of electrospinning‐based nanofibers materials, which will stimulate more work to promote the practical application of Li–S batteries.

## Conflict of Interest

The authors declare no conflict of interest.
